# Unique true predicted neoantigens (TPNAs) correlates with anti-tumor immune control in HCC patients

**DOI:** 10.1186/s12967-018-1662-9

**Published:** 2018-10-19

**Authors:** Annacarmen Petrizzo, Maria Tagliamonte, Angela Mauriello, Valerio Costa, Marianna Aprile, Roberta Esposito, Andrea Caporale, Antonio Luciano, Claudio Arra, Maria Lina Tornesello, Franco M. Buonaguro, Luigi Buonaguro

**Affiliations:** 1 Laboratory of Cancer Immunoregulation, Istituto Nazionale per lo Studio e la Cura dei Tumori, “Fondazione Pascale”-IRCCS, Via Mariano Semmola, 1, 80131 Naples, Italy; 2 Institute of Genetics and Biophysics “A. Buzzati-Traverso” (IGB), National Research Council, 80131 Naples, Italy; 3 Institute of Biostructures and Biomaging (IBB), National Research Council, 80134 Naples, Italy; 4 Animal Facility, Istituto Nazionale per lo Studio e la Cura dei Tumori, “Fondazione Pascale”-IRCCS, 80131 Naples, Italy; 5 Laboratory of Molecular Biology and Viral Oncology, Istituto Nazionale per lo Studio e la Cura dei Tumori, “Fondazione Pascale”-IRCCS, 80131 Naples, Italy

**Keywords:** Liver cancer, Immunotherapy, Cancer vaccine, Personalized treatment, Neoantigens

## Abstract

**Background:**

A novel prediction algorithm is needed for the identification of effective tumor associated mutated neoantigens. Only those with no homology to self wild type antigens are true predicted neoantigens (TPNAs) and can elicit an antitumor T cell response, not attenuated by central tolerance. To this aim, the mutational landscape was evaluated in HCV-associated hepatocellular carcinoma.

**Methods:**

Liver tumor biopsies and adjacent non-tumor liver tissues were obtained from 9 HCV-chronically infected subjects and subjected to RNA-Seq analysis. Mutant peptides were derived from single nucleotide variations and TPNAs were predicted using two prediction servers (e.g. NetTepi and NetMHCstabpan) by comparison with corresponding wild-type sequences, non-related self and pathogen-related antigens. Immunological confirmation was obtained in preclinical as well as clinical setting.

**Results:**

The development of such an improved algorithm resulted in a handful of TPNAs despite the large number of predicted neoantigens. Furthermore, TPNAs may share homology to pathogen’s antigens and be targeted by a pre-existing T cell immunity. Cross-reactivity between such antigens was confirmed in an experimental pre-clinical setting. Finally, TPNAs homologous to pathogen’s antigens were found in the only HCC long-term survival patient, suggesting a correlation between the pre-existing T cell immunity specific for these TPNAs and the favourable clinical outcome.

**Conclusions:**

The new algorithm allowed the identification of the very few TPNAs in cancer cells, and those targeted by a pre-existing immunity strongly correlated with long-term survival. Only such TPNAs represent the optimal candidates for immunotherapy strategies.

**Electronic supplementary material:**

The online version of this article (10.1186/s12967-018-1662-9) contains supplementary material, which is available to authorized users.

## Background

Cancer genome instability leads to accumulation of mutations which may result into tumor-specific mutated “neoantigens”, not be affected by central T cell tolerance [1, 2]. Therefore, neoantigens are considered the optimal target for the patient’s anti-tumor T cell immunity as well as for personalized cancer immunotherapy strategies [3, 4]. Naturally occurring tumor infiltrating lymphocytes (TILs) are mostly directed towards neoantigens, their frequency correlates to the tumor mutational burden and associate with increased survival [5–8]. Therapeutic cancer vaccines based on mutated neoantigens are currently evaluated in clinical trials showing significant prolonged patients’ survival, including also synthetic long peptide vaccines which are capable of inducing both CD8+ and CD4+ neoantigen-specific T-cell responses [9–12]. Moreover, clinical benefit of melanoma and lung cancer patients treated with checkpoint inhibitors (anti-CTLA-4 and PD-1) has been found to correlate not only with pre-existing T-cell infiltration and PD-L1 expression in tumors [13], but also with the mutational burden of treated tumors [14, 15].

However, experimental evidences show that neoantigens are not all the same. Indeed, they may show homology to wild type self antigens, becoming “invisible” to the immune system due to central tolerance mechanisms [16–18]. In such conditions, the tumor is more fitted to grow and less responsive to therapy with checkpoint inhibitors. Alternatively, they may show homology to pathogen-derived peptides, representing a more efficient target for a pre-existing T cell memory against such pathogens. In support of this, the latter type of neoantigens have been identified in cancer patients either long-term survivors [19] or responders to checkpoint inhibitors [15, 16].

Hepatocellular carcinoma (HCC) is the third leading cause of death from cancer globally. Therapeutic strategies for HCC are not very efficient and immunotherapies (i.e. cancer vaccines) can help in improving clinical outcome [20–22]. Among others, HCC ranks as a medium-variable tumor, with an average mutational burden of 5 somatic mutations per Mb, corresponding to approximately 60 non-synonymous substitutions within expressed genes [23]. Most of the reported data on the mutational landscape of HCC have been generated on samples associated with HBV chronic infection and are based on exome sequencing analyses [24–26]. Nevertheless, none of the studies explored the correlation between somatic mutations and the repertoire of HCC-specific “neoantigens”.

Here we describe an improved algorithm for the prediction and validation of mutated neoantigens in HCV-associated HCC. Indeed, HCV is the most frequent risk factor for HCC worldwide, together with HBV. Our finding show that true neoantigens (TPNAs) are only those which do not share sequence homology with any self antigens. Moreover, long-term survival correlates with the identification in the tumor of TPNAs homologous to pathogen-derived peptides.

Our experimental clinical evidences show that the quality more than the quantity of neoantigens expressed by cancer cells may predict clinical outcome as well as guide selection of the most appropriate target antigens for cancer immunotherapy.

## Methods

### Patients and tissue samples collection

Nine HCV chronic infected HCC patients undergoing liver resection were enrolled for the present study (Additional file 1: Table S1). Two paired liver biopsies from each patient with histologically-confirmed HCC and adjacent non-tumor liver tissue were obtained at the time of surgery and stored in RNA-stabilizing agent (RNAlater, Qiagen) for RNA sequencing. Peripheral blood was obtained by venipuncture from the HCC patient HLA-008. All human specimens were obtained and processed at the National Cancer Institute in Naples under informed consent. Fresh human peripheral blood mononuclear cells (PBMCs) were isolated by Ficoll-Hypaque density gradient centrifugation and cultured in appropriate medium consisting of RPMI 1640 (Capricorn Scientific GmbH) containing 2 mM l-Glut, supplemented with 10% heat inactivated human serum (Capricorn Scientific GmbH), 25 mM HEPES buffer solution (Capricorn Scientific GmbH), 50 IU/ml penicillin and 50 μg/ml streptomycin (Gibco Life Technologies), 20 μg/ml gentamicin (Capricorn Scientific GmbH). PBMCs were maintained at 37 °C in a humidified incubator with 5% CO_2_.

### Cell line

Human TAP-deficient T2 cell line (174× CEM.T2; ATCC CRL-1992™) was purchased from American Type Culture Collection (ATCC; https://www.atcc.org/). T2 cell line was maintained in Iscove’s modified Dulbecco’s medium (IMDM; Gibco Life Technologies) containing 25 mM HEPES and 2 mM l-Glut, supplemented with 20% fetal bovine serum (FBS; Capricorn Scientific GmbH), 100 IU/ml penicillin and 100 μg/ml streptomycin (Gibco Life Technologies). Cells were maintained at 37 °C in a humidified incubator with 5% CO_2_.

### RNA extraction, library preparation and sequencing

Liver samples were homogenized in TRIzol reagent using the Tissue Lyser Disruption system (Qiagen), flash frozen on dry ice, and total RNA was purified according to manufacturer’s protocol (Invitrogen). RNA samples were quantified by NanoDrop1000 spectrophotometer (Thermo Fisher Scientific) and displayed a 260/280-absorbance ratio about 1.8–2. RNA quality was assessed by digital electrophoresis on Experion System by RNA StdSens Kit and RNA chips (Bio-Rad). Preparation of sequencing cDNA libraries was carried out on 4 μg of total RNA per sample using the TruSeq RNA stranded Sample Preparation Kit (Illumina). Paired-end cDNA libraries were quantified by Qubit Fluorometer (Q32866; LifeTechnologies) and Qubit dsDNA High Sensitivity Assay Kit. The overall quality of the libraries was evaluated on Experion System by DNA 1 K Analysis Kit and DNA chips (Bio-Rad). Paired-end libraries (100× 2 bp) were sequenced at high coverage on the Illumina HiSeq 2000 NGS [27].

### Analysis of RNA-seq data

Quality control check was performed on the total number of raw reads using FastQC tool (http://www.bioinformatics.babraham.ac.uk/projects/fastqc/). High-quality reads were mapped to human reference transcriptome and to human reference genome using TopHat2 v2.0.10 [28]. Only uniquely mapped reads were used to quantify gene expression in each sample and to compute differential expression between non-tumour and tumour samples [29]. Principal component analysis (PCA), MA and density plots for an overall control of the experiment were generated using RNASeqGUI. Differential expression between tumour and adjacent non-tumour samples was evaluated using the GLM implemented in EdgeR. FDR < 0.01 was used as threshold to determine differentially expressed genes (DEGs).

The analysis of pathways and gene ontology for the DEGs was performed using DAVID, both implemented in the GUI. Multiplicity correction (false discovery rate, FDR) was used for enrichment of DEGs, with a threshold of 0.05.

For the SNP calling, PCR duplicates were removed using Picard tools v1.117 and SNP calling was carried out using GATK v3.3 workflow optimized for RNA-seq reads. Nucleotide variants were then annotated using ANNOVAR. Common variants reported in dbSNP (v138), in the 1000 genome database, as well as those identified in adjacent non-tumor liver tissue were filtered out from our dataset. SNVs in super-duplicated regions were also removed.

### Epitope prediction and sequence homology analysis

Epitope prediction was performed for non-synonymous somatic SNVs using prediction tools available at http://www.cbs.dtu.dk/services/.

The NetTepi version 1.0 as well as NetMHCstabpan version 1.0 were used to predict MHC class I HLA-A*02:01 allele restricted epitopes. A peptide sequence of 30 amino acid, centered on the mutated amino acid, was used to predict 9-mer neoantigens scanning the entire sequence by overlapping peptides. In both servers, epitopes were predicted based on the combination of different parameters and ranked on prediction values (%rank). Predicted epitopes were selected based on %rank ≤ 2. The Immune Epitope Database (IEDB; http://www.iedb.org/) was used for analysis of sequence homology to experimentally validated human and pathogen-derived antigens. Known antigens with homology > 70% to mutated antigens were identified, but only those with matching aa residues at the TCR binding positions (positions 1, 4, 5 and 8) were selected for subsequent analyses. Homologous validated antigens were subsequently confirmed by the NetMHCstabpan version 1.0.

### Peptide synthesis

Peptides were synthesized at a purity > 95%. Lyophilized powder was dissolved in dimethylsulfoxide (DMSO; Sigma-Aldrich), diluted in phosphate-buffered saline (1× PBS; Gibco Life Technologies) and stored at − 80 °C until use.

### Peptide binding affinity and BFA decay assays

Peptide binding affinity to HLA-A*02:01 molecule and BFA decay assays were performed for each candidate peptide. Briefly, T2 were seeded at 3.5 × 10^5^ cells per well in 24-well plates and incubated overnight with peptides (final concentrations: 10 μM, 20 μM, 50 μM, 100 μM) in IMDM serum-free medium containing 3 μg/ml human β2-microglobulin (Sigma-Aldrich) in a humidified incubator at 37 °C with 5% CO_2_. Following incubation, cells were harvested and centrifuged at 200×*g* for 5 min. Subsequently, cells were washed twice with phosphate-buffered saline (1× PBS; Gibco Life Technologies) and stained with R-PE conjugated anti-human HLA-A2 monoclonal antibody (cat. 343306; BioLegend), for 30 min at 4 °C, and analyzed with the Attune™ N×T flow cytometer (Thermo Fisher Scientific). OVA peptide was used as negative control and T2 cells without any added peptide were used as a background control. A fluorescence index (FI) was calculated using the following formula: FI = [mean fluorescence intensity (MFI) sample − MFI background]/MFI background, where MFI background represents the value without peptide. An FI > 0.5 was set as threshold to indicate peptides with affinity for the HLA-A*02:01 molecule. For the brefeldin A decay assay, T2 were seeded at 5 × 10^5^ cells per well in 24-well plates and cultured overnight with either the candidate peptides or the control peptide (CAP-1 was used as control) at a final concentration of 50 μM, at 37 °C in a humidified incubator with 5% CO_2_, in serum-free IMDM medium containing 3 μg/ml β2-microglobulin (Sigma-Aldrich). Following incubation, cells were washed and incubated with 1× BFA (brefeldin A solution; cat. 420601; BioLegend) in IMDM serum-free medium, for 1 h at 37 °C. Cells were harvested every 2 h (T0, T2, T4, T6, T8), washed with phosphate-buffered saline (1× PBS; Gibco Life Technologies), stained with anti-HLA-A*0201 fluorescent monoclonal antibody (cat. 343306; BioLegend) and analyzed by flow cytometry. The stability of each peptide bound to HLA-A2 was measured as the DC_50_ value, which was defined as an estimate of the time required for a 50% reduction of the MFI value recorded at time 0. The DC_50_ value was calculated according to the formula: MFI at indicated time points/MFI at time 0 × 100. All the experiments were performed in triplicate.

### In vivo immunization

C57BL/6 (H-2b MHC) female mice, 8 week old, were purchased from Harlan (Udine, Italy). All animals were housed at the Animal Facility of the Istituto Nazionale Tumori “Pascale” (Naples,Italy). Mice were housed in number of 2–3 per cage and maintained in a conventional facility on a 12 h light:12 h dark cycle (lights on at 7:00 a.m.) in a temperature-controlled room (22 ± 2 °C) and with food and water ad libitum at all times. The experimental protocols were in compliance with the European Communities Council directive (86/609/EEC) and were approved by the Italian Ministry of Health (approval number 835/2016).

Animals (6 for each group) were injected by sub-cutaneous route with 100 µg of each peptide, emulsified with 50 μg of Polyinosinic:polycytidylic acid [poly(I:C); InvivoGen] adjuvant formulated in PBS (200 μl total volume). Immunization was performed twice a week for a total of six administrations. At the time of sacrifice, spleens were resected and processed into single cell suspensions using a gentle MACS Dissociator (Miltenyi Biotec) according to the manufacturer’s instructions for immunological evaluation.

### IFN-γ ELISpot

IFN-γ ELISpot (BD™ human IFN-γ ELISPOT Set) assay was performed on splenocytes from the immunized mice as well as PBMCs from the HLA-008 HCC patient.

For the splenocytes from immunized mice, 2 × 10^5^ splenocytes pooled from animals in each group were counted and plated in each well. Cells were stimulated with 10 μg/ml of single and pool peptide used for the immunization and incubated for 24–26 h. As negative and positive control, peptide diluents PBS and 10 μg/ml of PHA (PHA-K; Capricorn Scientific GmbH) were used respectively.

For the PBMCs from the HCC patient and healthy donors, 4 × 10^6^ PBMCs/ml/well were stimulated with peptides at a final concentration of 10 μg/ml. On day three, 10 U/ml IL-2 was added to each well. On day five, half of the volume of medium was replaced with fresh medium containing IL-2 at a final concentration of 10 U/ml. On day seven, PBMCs were re-stimulated with each peptide. On day 10, cells were harvested for IFN-γ ELISpot assay. Each peptide was added at a final concentration of 10 μg/ml to 2 × 10^5^ PBMCs per well in 100 μl RPMI 1640 medium (Capricorn Scientific GmbH). PBMCs were cultured at 37 °C in a humidified incubator with 5% CO_2_ for 20 h. Stimulation with 10 μg/ml PHA (PHA-K; Capricorn Scientific GmbH) was used as positive control, PBMCs without added peptides were used as the negative control, RPMI 1640 medium (Capricorn Scientific GmbH) was used as background control.

The plates were read with an AID EliSpot Reader Systems (AID GmbH, Strassberg, Germany). Determinations from triplicate tests were averaged. Data were analyzed by subtracting the mean number of spots in the wells with cells and medium-only from the mean counts of spots in wells with cells and antigen. Spot forming units (SFU) were calculated as the frequency per 10^6^ PBMCs.

### Statistical analysis

Comparison between individual data points were performed with the unpaired two-sided Student’s t-test and ANOVA, as appropriate. Normally distributed data were represented as mean ± SEM. All p values were two-tailed and considered significant if less than 0.05.

## Results

### HCC patients’ characteristics

Nine HCC patients were enrolled for the present study. They were all HCV chronically infected, with an average age of 72.8 years old. They were all positive for the HLA-A*02:01 haplotype and only two (HLA-008 and HLA-026) were positive also for the HLA-A*24:02 haplotype. Three patients (HLA-008, HLA-009 and HLA-012) are still alive at the moment of submission, but only HLA-008 is still living in the area (Additional file 1: Table S1).

### Transcriptome analysis of HCV-related HCC

Samples from paired primary HCV-related HCC and non-tumour adjacent liver tissues were subjected to RNA-seq analysis, and a clear segregation of gene expression values in tumor and the adjacent liver tissues was observed (Additional file 2: Fig. S1A, B).

**Fig. 1:**
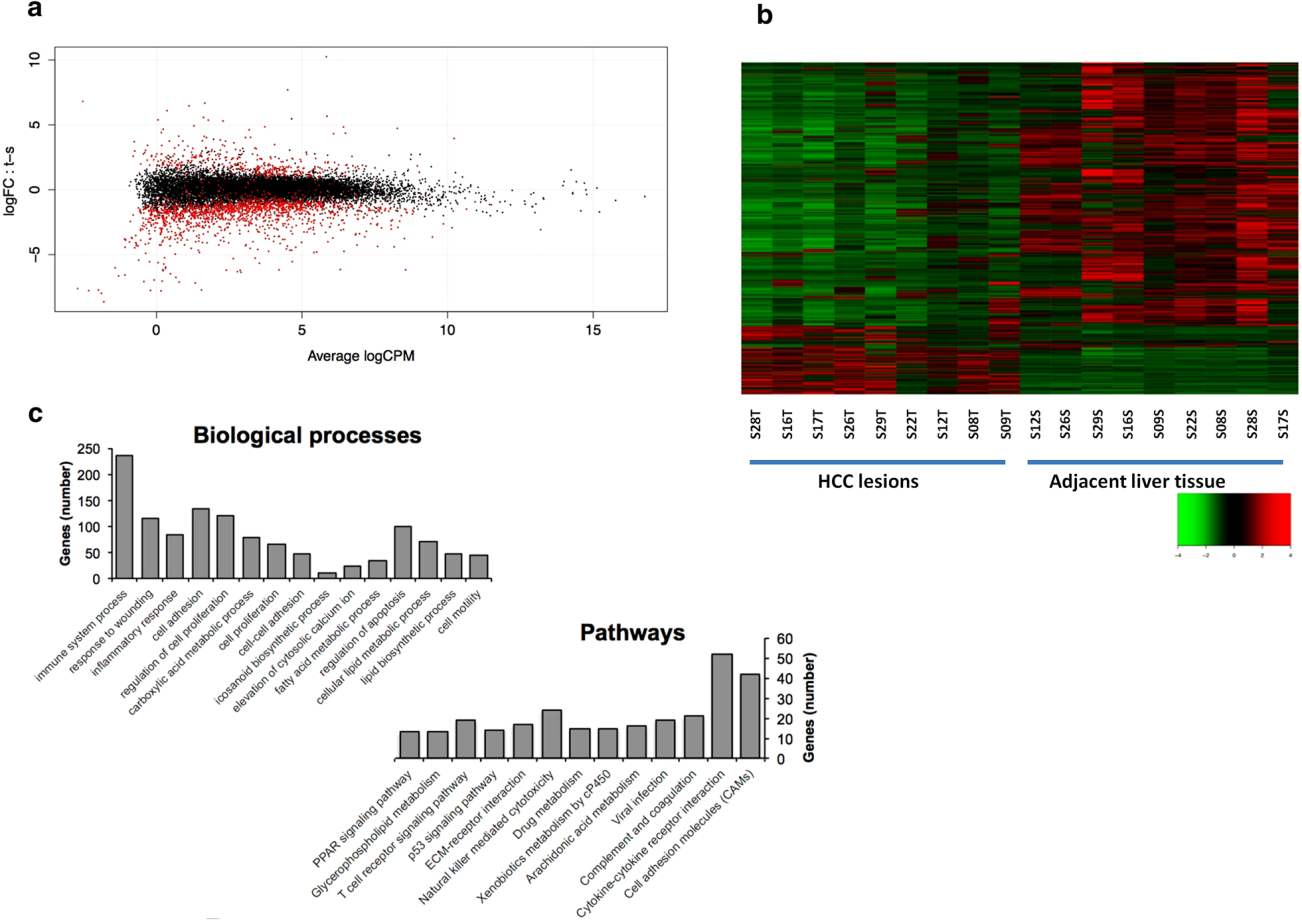
Gene expression analysis of NCV-related HCC samples. Differential expression of 2101 genes in HCC and adjacent liver tissues represented by MD plot (**a**) and heatmap (**b**). List of biological processes and pathways whose genes are modulated in HCC tissues. The number of modulated genes in each process and pathway are indicated on the y axis (**c**)

The expression of 2101 protein-coding genes was modulated in the HCC samples vs. the corresponding non tumour adjacent tissues, with a degree of differential expression ranging from − 8.35 to 8.98 log2FC (FDR < 0.01; Fig. 1a). Most of differentially expressed genes (DEGs) (about 78%, i.e. 1639 genes) were significantly down-modulated in HCC samples, suggesting a strong gene transcription silencing in HCC lesions (Fig. 1b). A significant alteration of 15 biological processes and 13 pathways, such as cell adhesion molecules, viral infection and drug metabolism pathways (Bonferroni adjusted *p* values ≤ 0.05), was observed (Fig. 1c). Notably, PI3K, Wnt, p53 and Ras signaling pathways were significantly altered in HCV-related HCC vs non-tumor adjacent tissues (Additional file 2: Fig. S2A–D).

### Selection of mutations for identification of potential neoantigens in HCC

In order to identify potential neoantigens specific for HCV-related HCC, reads generated by RNA-seq were used for the SNP calling. More than forty-thousand Single Nucleotide Variations (SNVs) were found in the exons of our samples, of which more than 50% were non-synonymous SNVs (nsSNVs). 1516 potentially somatic mutations in 1357 genes were selected, based on criteria described in Materials and methods (Additional file 2: Fig. S3A). The number of mutations identified in each sample are reported in Additional file 2: Fig. S3B. As expected, the largest fraction was constituted by nsSNVs and about 25–30% of them were predicted to be deleterious to the protein functionality (Additional file 2: Fig. S3C).

We then focused only on the 1100 nsSNVs found in a set of 249 genes highly expressed (4th quartile) both in HCC and matched non-tumor liver tissues. The vast majority of genes bearing the selected mutations were not shared among the HCC samples. Only in few cases, the same gene was mutated in different HCC samples but the observed nsSNVs were always distinct (Additional file 1: Table S2).

### MHC class I epitope prediction

All the 1100 mutations nsSNVs were evaluated for MHC class I HLA-A*02:01 allele-restricted neoantigen prediction according to the NetTepi and NetMHCstabpan parameters described in “Methods” section.

**Fig. 2:**
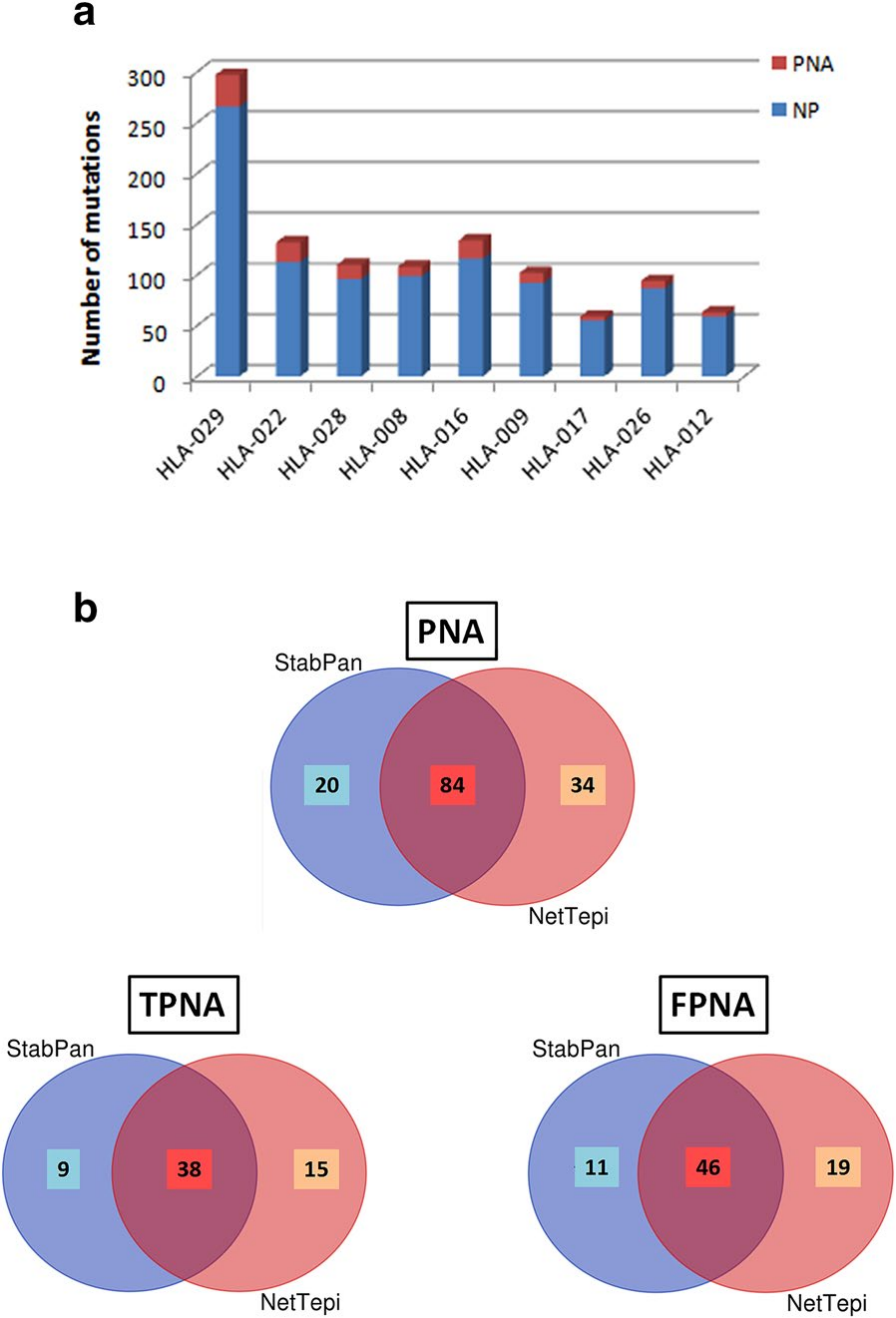
Neoantigen prediction in HCC samples. Neoantigens were predicted by NetTepi and NetMHCstabpan algorithms from the 1100 nsSNVs identified in 249 highly modulated genes. **a** Number of non-predicted (NP) and predicted neoantigen (PNA) in each HCC sample; **b** Venn diagrams showing the number of common and unique PNAs, TPNAs and FPNAs predicted by the NetTepi and NetMHCstabpan servers

The prediction analysis was performed with the two servers running a side-by-side comparison between paired wild-type and mutated peptide sequences. According to such analysis, in each sample most of the mutations were not predicted to be neoantigens (NP) (Fig. 2a). According to the default threshold of total %rank ≤ 2, the two servers identified in each samples predicted neoantigens (PNAs) which overlapped in the majority of cases (Additional file 2: Fig. S4). Overall, the NetTepi server predicted 118 neoantigens and the NetMHCstabpan predicted 104 neoantigens, of which 84 were common (Fig. 2b). Considering only the neoantigens predicted by both servers, the average number of PNAs in the HCC samples was 9.33, ranging from 19 (HLA-029) to 2 (HLA-012) (Additional file 2: Fig. S5). However, 46 of the 84 PNAs (71.5%) should be considered as “false” predicted neoantigens (FPNAs) given that their corresponding wt peptides are characterized by a similar predicted antigenicity (e.g. both with a %rank ≤ 2) (Additional file 1: Table S3). Therefore, only 38 PNAs are “true” predicted neoantigens (TPNAs) in the whole set of HCC samples, given that their corresponding wt peptides are not predicted to be antigenic (Fig. 2b; Additional file 2: Fig. S6). The average number of TPNAs in the HCC samples was 4.22, ranging from 7 (HLA-008 and 022) to 1 (HLA-012) (Additional file 2: Fig. S5).

### In silico analysis of false predicted neoantigens (FPNAs)

In order to closely analyze the “false” PNAs, the sequences of FPNAs were aligned to the corresponding wild type peptides. According to previous findings [30], we compared the aminoacid residues at positions p1, p4, p5 and p8, which are exposed to the T cell receptor (TCR) when epitopes are allocated in the HLA groove. The analysis confirmed that 24 of the 46 (52.2%) FPNAs show all 4 aa residues identical to the corresponding wild type epitope; the remaining FPNAs (47.8%) show homology in 3/4 aa residues (Additional file 2: Fig. S7).

### In vitro analysis of TPNAs binding affinity and stability to HLA-A*0201 molecule

**Fig. 3:**
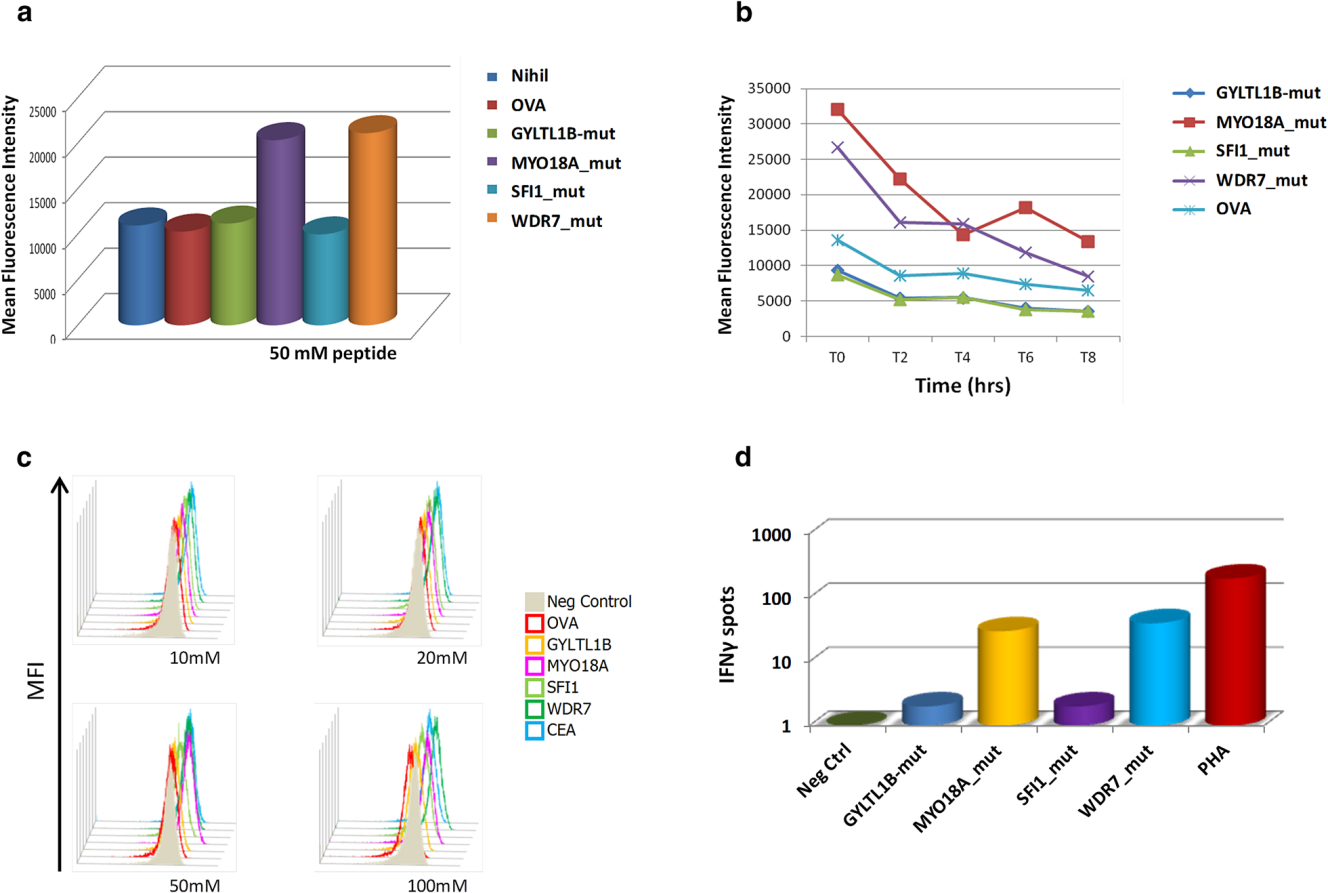
Experimental binding of TPNAs to HLA-A*0201. Binding to HLA-A*0201 molecule and relative stability was assessed in TAP-deficient T2 cells loaded with the indicated peptides. **a** Mean fluorescence intensity at flow cytometer indicates binding levels of peptides to HLA molecules. **b** Decay of mean fluorescence intensity over time indicates stability of the peptide binding to the HLA molecule. MFI_50_ measures the stability of the binding expressed in hours. **c** Overlay of the mean fluorescence intensity observed with different concentration of peptides. **d** PBMCs isolated from HLA-A*02:01 healthy donor were stimulated ex vivo with the indicated peptides for 10 days. Interferon-γ (IFN-γ) secreting T cells were evaluated after in vitro O/N restimulation with individual peptides

In order to experimentally confirm the binding and stability of TPNAs to HLA-A*0201 molecule, the HLA-A*02-positive T2 cell line was loaded with 10 TPNAs with high (< 0.8 in NetTepi and < 1.3 in StabPan) and low (0.8 < %Rank < 2 in NetTepi and 1.3 < %Rank < 2 in StabPan) prediction %rank. The analysis showed that only the peptides with high prediction %rank (MYO18A_mut and WDR7_mut) induced a significant increase in the HLA surface expression on T2 cells over background as well as OVA negative control level (Fig. 3a).

The peptide—MHC dissociation kinetics showed that, independent of the %rank, all peptide-MHC complexes showed a 50% dissociation value between T4 and T6, although with different MFI values for the different peptides (Fig. 3b).

Consequently, selecting only the TPNAs with the high prediction %rank in both servers, the overall number of high affinity and stability TPNAs dropped down to nine, with some of tumour samples (e.g. HLA-012, 022, 026) showing no predicted ones (Additional file 1: Table S4). As predictable, similar results have been obtained using FPNAs with different prediction %rank, confirming that only epitopes with high prediction values show binding to HLA molecules. On the contrary, none of the not predicted neoantigens (NP) showed binding to HLA molecules (data not shown). To further confirm the antigenicity of such predicted neoantigens, they were used to stimulate ex vivo also PBMCs from HLA-A*02:01 healthy donors. Results confirmed that only the peptides with high prediction %rank (MYO18A_mut and WDR7_mut) were able to induce release of IFNγ in PBMCs (Fig. 3d).

### Analysis of binding motifs and profiles of TPNA sequences

In order to identify possible consensus patterns in the TPNAs aminoacid sequences, the TPNA sequences were aligned all together or divided in high (< 0.8 in NetTepi and < 1.3 in StabPan) and low (0.8 < %Rank < 2 in NetTepi and 1.3 < %Rank < 2 in StabPan) (9 and 44 sequences, respectively). As predictable, amino acid residues at the HLA binding positions p2 and p9 showed the highest conservation across sequences, and only minor differences were observed in the three different alignments (Additional file 2: Fig. S8). On the contrary, the TCR binding positions p1, p4, p5, p8 showed a significant variability with no prevalent aminoacid residue. However, the 9 TPNAs with highest %rank showed a profile at these positions significantly different from the one observed in the other two alignments (Additional file 2: Fig. S8A–C). In particular, at positions p1, p4 and p8, it was observed a prevalence of glycine (G) instead of leucine (L) (Additional file 2: Fig. S8D–F).

### Sequence homology analysis with known epitopes

In order to definitely confirm that the TPNAs are indeed neoantigens and do not share homology with any known antigen reported in the literature, a blast search was run against epitopes in the Immune Epitope Database (IEDB; http://www.iedb.org/).

According to such analysis, 31 TPNAs showed a sequence homology to known human and/or pathogen-derived epitopes (Additional file 1: Tables S5, S6). However, a prediction analysis with NetTepi confirmed that only 4 human self epitopes and 9 pathogen-derived epitopes were associated to the HLA-A02*01 haplotype. Of these, considering the homology at TCR binding positions, only 3 predicted human self epitopes and 3 pathogen-derived epitopes could really be considered homologous to the corresponding TPNAs (Additional file 2: Fig. S9). Interestingly, the sample HLA-008 was the only one showing more than one TPNA homologous to known antigens. In particular, it showed 3/7 TPNAs homologous to pathogen-derived epitopes (e.g. Lassavirus, Shigella and Vaccinia virus) without homology to any cellular self epitope.

### Validation of immunological homology between TPNAs and known epitopes in a mouse model

In order to prove that the TPNAs and their homologous known epitopes were able to elicit a cross-reactive immune response, an in vivo immunization experiment was performed in a C57BL/6 mouse model. Animals were independently immunized with the HLA-008 TPNA CHD3_mut or its homologous Shigella epitope, and with the HLA-017 TPNA NOP2_mut or its homologous human self epitope DDIT3. All such epitopes, indeed, bind to human HLA-A*0201 as well as to mouse H-2-Db/Kb haplotypes. However, while the CHD3_mut and the homologous Shigella epitopes are predicted to bind both H-2-Db and H-2-Kb haplotypes, the NOP2_mut and the homologous DDIT3 epitopes are predicted to bind only the H-2-Kb haplotype (Additional file 1: Table S7).

**Fig. 4:**
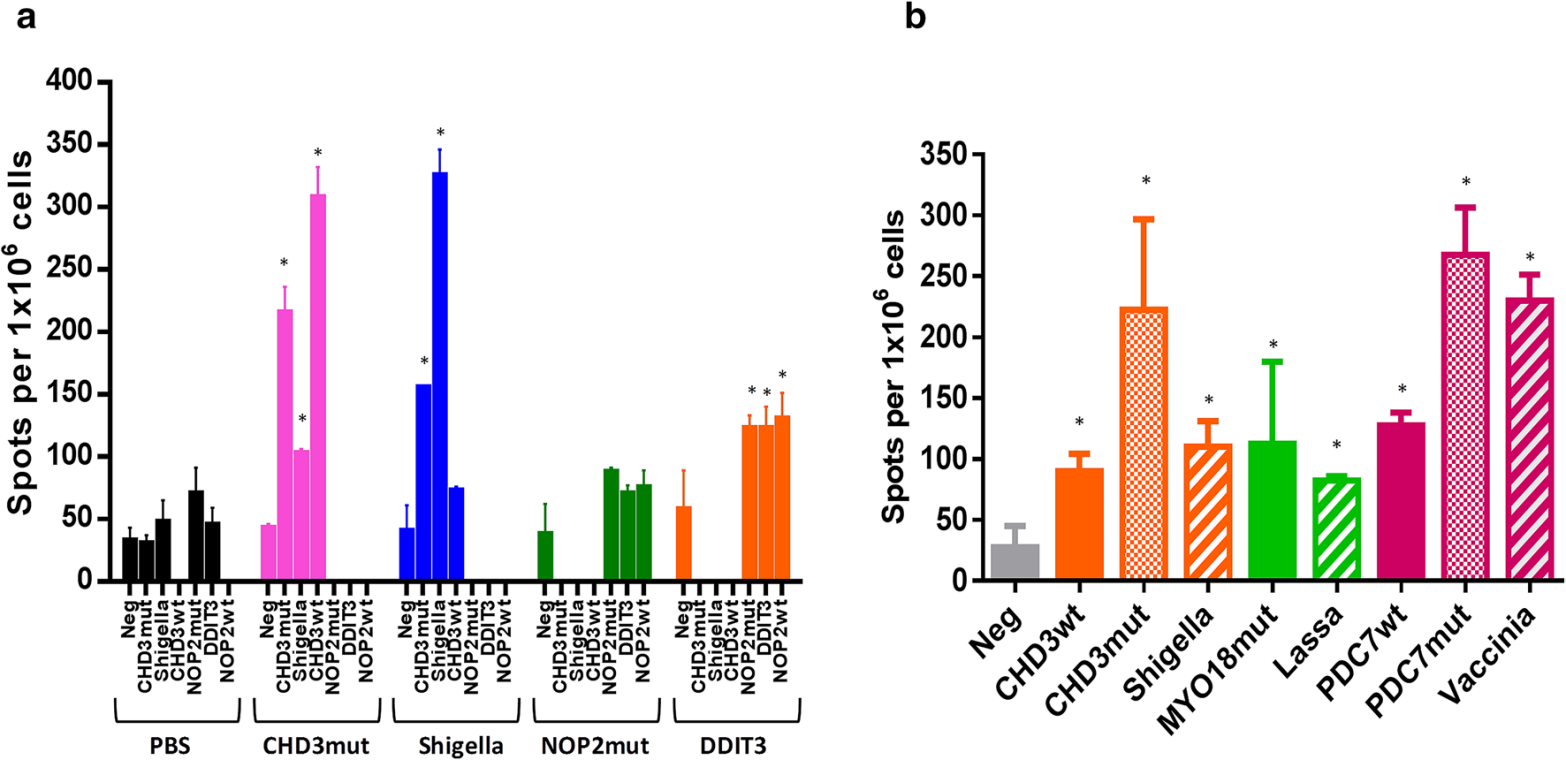
Evaluation of pre-existing immunity to TPNAs in a long-term survivor. **a** C57BL/6 mice were immunized with the indicated peptides or with PBS. Interferon-γ (IFN-γ) secreting T cells were evaluated in splenocytes from sacrificed animals after in vitro O/N re-stimulation with individual peptides. **b** PBMCs isolated from patient HLA-008 were stimulated ex vivo with the indicated peptides for 10 days. Interferon-γ (IFN-γ) secreting T cells were evaluated after in vitro O/N restimulation with individual peptides

The results confirmed that the CHD3_mut and the homologous Shigella epitopes induced a much stronger immune response than the NOP2_mut and the homologous DDIT3 epitopes. Moreover, as predicted by the bioinformatics algorithm, PBMCs from animals immunized with the TPNA CHD3_mut and the homologous Shigella epitope, respectively, cross reacted against both epitopes. The reactivity against the homologous epitope was about 50% of the one observed against the peptide used for the immunization (Fig. 4a). Such a result can be due to the mismatch in the alignment of the residues at the TCR binding positions (Additional file 2: Fig. S9). As predictable, PBMCs from animals immunized with the TPNA CHD3_mut showed stronger reactivity to CHD3_wt than to Shigella due to the 100% homology in the aa residues at the TCR binding positions. Similar results, but at much lower intensity, were obtained in mice immunized with the NOP2_mut and the homologous DDIT3 epitopes (Fig. 4a).

### Correlation between pre-existing immunity to pathogen-derived epitopes and response to TPNAs in the long-term HCC survivor

In order to verify whether the cross reactive immunity between pathogen-derived epitopes and TPNAs correlates with improved survival, PBMCs from the only HCC patient still surviving to date (HLA-008) were obtained. The results clearly showed that the patient had variable levels of circulating T cells reacting to all three TPNAs as well as to the pathogen-derived epitopes. However, the two most reactive peptides were the TPNA PDCD7_mut and its homologous Vaccinia virus epitope, strongly suggesting that the pre-existing memory T cell response induced by the vaccinia virus vaccination may favor a stronger reactivity against the tumor-specific neoantigen (Fig. 4b). Interestingly, the T cell reactivity against the CHD3_mut peptide was of the same magnitude as for the PDCD7_mut peptide, although the levels of pre-existing memory T cell response against the homologous Shighella epitope was significantly lower (Fig. 4b). On the contrary, the low reactivity to the MYO18_mut did not reflect the predicted high antigenicity and stability and was not boosted by the low levels of pre-existing memory T cell response specific for its homologous epitope derived from Lassavirus. Finally, as predictable by the lack of matching in the TCR binding positions, no cross-reactivity was observed between the CWF19L1_mut and hepatitis C virus epitope (data not shown).

### Analysis of the tumor immune microenvironment in the HCC samples

The intratumor immune landscape for each of the HCC samples was assessed evaluating the transcription levels of immune-related genes. In particular, infiltration of effector and regulatory cell types as well as expression of immune checkpoint and antigen presenting molecules were evaluated by such analysis. The immunophenotype (IP) for each HCC patient was generated using the online tool available at The Cancer Immunome Atlas (https://tcia.at/). A more pronounced immune effective microenvironment, characterized by higher gene expression of effector cell types and antigen presenting molecules, gives a higher IP score [31].

**Fig. 5:**
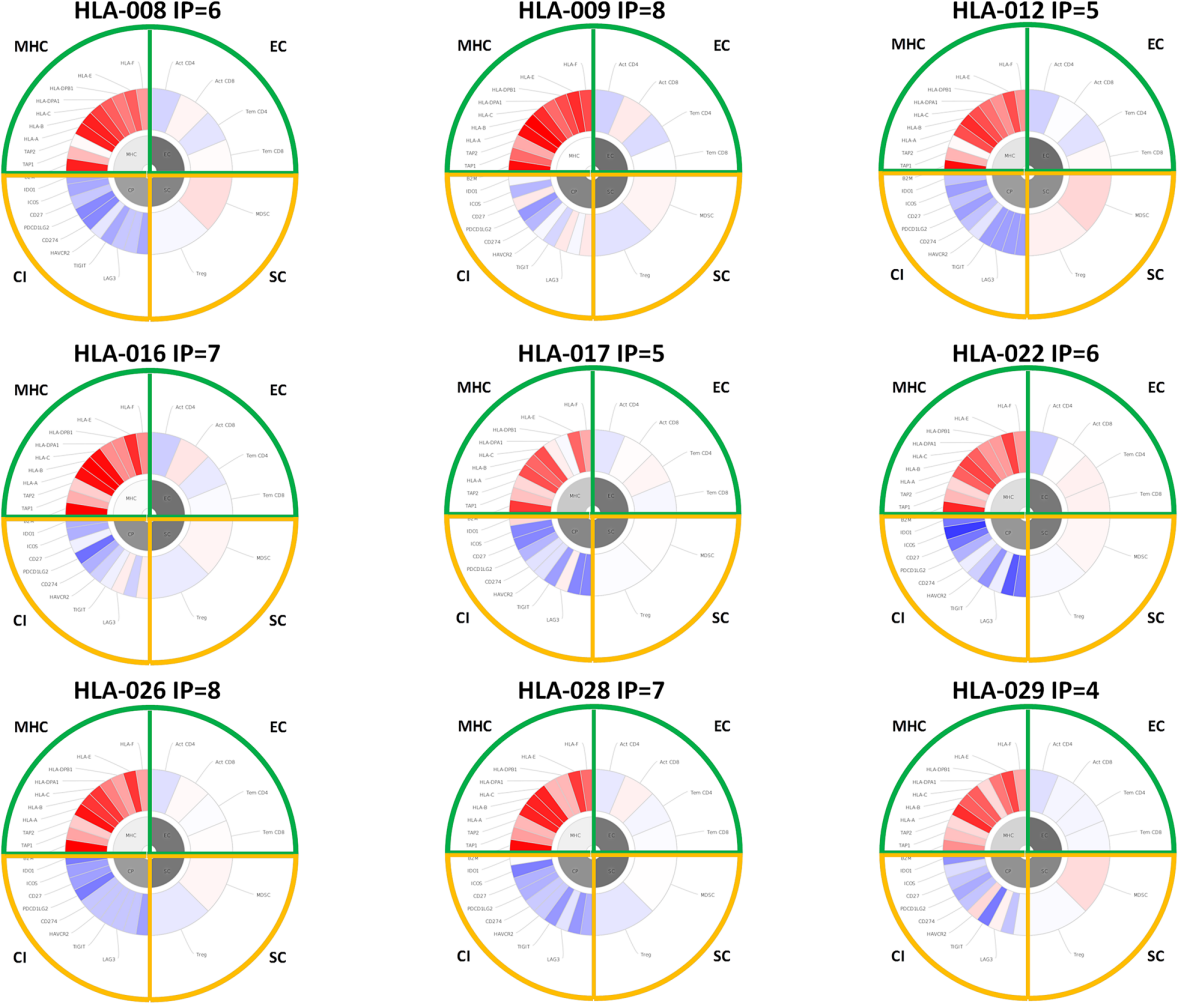
Assessment of the immunophenogram for each HCC sample. Expression values for immunologically related genes were used to generate the immunophenogram for each sample. In particular, genes included in the analysis were representative of Effector Cells (Activated CD4+ Tcells; Activated CD8+ Tcells; Memory CD4+ Tcells; Memory CD8+ Tcells); Suppressor Cells (MDSC; Tregs); Checkpoint inhibitors; HLA molecules. The list of genes included in the analysis and characterizing the individual immune cell populations is at http://www.iedb.org

The results showed that, with the exception of the HLA-012, HLA-017 and HLA-029 with an IP < 6, all other HCC samples had an IP ≥ 6 (Fig. 5), which has been reported to be associated with clinical benefit and/or long-term survival. Interestingly, the long-term survivors HLA-008 and HLA-009 showed an IP ≥ 6, supporting the correlation between the immune determinants included in the immunophenogram and the clinical outcome.

## Discussion

Samples from nine paired primary HCV-related HCC and non-tumour adjacent liver tissues were collected for RNA-seq. The expression level of 2101 protein-coding genes was found to be specifically modulated in HCV-related HCC (FDR < 0.01), with a significant alteration of several pathways, including cell adhesion molecules, viral infection and drug metabolism, PI3K, Wnt, p53 and Ras signaling.

Prediction of HCC specific neoantigens was performed on a set of 249 genes highly expressed (4th quartile) both in HCC and matched non-tumor liver tissues. A prediction analysis by the combination of NetTepi and NetMHCstabpan servers, based on the default parameters, showed that only 84 of the 1100 (7.6%) nsSNVs were predicted to generate neoantigens by both servers. Considering only the neoantigens predicted by both servers, predicted neoantigens (PNAs) in the analyzed HCC samples were 9.33, ranging from 19 (HLA-029) to 2 (HLA-012). The finding that > 90% of the somatic nsSNVs are not predicted to generate neoantigens is likely the outcome of the tumor immunoediting operated by the immune system [32]. Indeed, after the elimination of highly immunogenic tumor cells presenting several neoantigens, only poorly immunogenic cancer cells, expressing very few or no neoantigens, may survive and outgrow to give rise to large tumor mass.

However, in order to be effectively recognized by T cells, PNAs must be immunogenically different from the corresponding wild type peptide. Consequently, a PNA can be defined as “true” (TPNA) only if the corresponding wild type peptide is not predicted to be an epitope. Alternatively, it should be considered as “false” PNA (FPNA) and, according to such a definition, more than 70% of the PNAs identified in the enrolled HCC patients were indeed FPNAs. The alignment of each FPNA to its corresponding wild type peptide confirmed that the amino acid residues at the position p1, p4, p5 and p8 showed a 100% homology (4/4 aa) in 24/46 pairs and 75% homology (3/4 aa) in the remaining 22/46 pairs. In fact, when the epitope is allocated in the groove of the HLA class I molecule, only such positions are exposed and interacting with the T cell receptor (TCR) [30]. Therefore, the observed homology at these residues makes the FPNA a “self” antigen able to escape the immunological control because the specific T cell clones have been removed by the central tolerance mechanisms.

The remaining TPNAs are the real target of the anti-cancer immune response and, in the present study, represented less than 3.45% of the all 1100 nsSNVs identified. In particular, they were 4.22 per each HCC sample on average, ranging from 7 (HLA-008 and 022) to 1 (HLA-012). However, the experimental validation of TPNAs clearly indicated that only those with a high %rank (< 0.8 in NetTepi and < 1.3 in StabPan) showed a significant binding to HLA class I molecule and stability. On the contrary, TPNAs with a prediction value 0.8 < %Rank < 2 in NetTepi and 1.3 < %Rank < 2 in StabPan did not show binding and stability significantly higher than control. Such an observation implies that only TPNAs with a high %Rank (< 0.8 in NetTepi and < 1.3 in StabPan) should be considered as efficient targets for anti-tumor T cell immunity. Based on such an evidence, only a total of 9 TPNAs were identified in the HCC samples, with some of them showing no prediction (e.g. HLA-012, 022, 026). Results obtained with our prediction algorithm are fully confirmed by the prediction based on the differential agretopicity (DAI) between mutant and wild type epitope originally described by Duan et al. (data not shown) [33].

The alignment of amino acid sequences of TPNAs showed a quite conserved pattern in the positions relevant for the binding to HLA, which is expected because they must all interact with the residues in the groove of the same HLA A*0201 molecule. Interestingly, considering the four TCR binding residues only, a significant variability was observed with no prevalent aminoacid on the others. However, the consensus sequence of TPNAs with highest %rank (< 0.8 in NetTepi and < 1.3 in StabPan) showed a prevalence of glycine (G) at positions p1, p4 and p8, instead of leucine (L). Obviously, such an intriguing observation requires additional studies on a much higher number of samples to confirm it and provide a possible immunological explanation.

We further characterized the predicted TPNAs by assessing homology with any known epitopes of human or pathogen origin. Indeed, if TPNAs share homology with an epitope of human origin, such TPNAs do not represent a target because they are immunogenically hidden “self” antigens allowing the tumor to escape the immunological control. On the contrary, if they share homology with an epitope of pathogen origin, such TPNAs may provide a selective advantage for the immune system to control the tumor growth. Indeed, if the patient has a pre-existing immunity for that specific pathogen-derived epitope, he will have the chance to respond sooner and stronger against the tumor. To this aim, a blast search in the Immune Epitope Database (IEDB; http://www.iedb.org/) showed that 31/53 TPNAs identified in the present study shared homology to known human and/or pathogen-derived epitopes. However, according to the homology at the TCR binding positions, only 3 “detrimental” human self epitopes and 3 “beneficial” pathogen-derived epitopes could be considered homologous to the corresponding TPNAs. Immune cross-reactivity between TPNAs and their homologous epitopes was confirmed in animals, proving that the homologous paired epitopes are recognized by the same T cell population. This implies that, depending on the homology to a “detrimental” or a “beneficial” epitope, TNPAs may have a completely different immunogenicity providing a totally different fate to the cancer cell.

Interestingly, the 3 homologous pathogen-derived epitopes were all identified in the long-term survivor HLA-008 patient. The relevance of the homology between the TPNAs and pathogen-derived epitopes in the favorable clinical outcome of the HLA-008 patient was confirmed by the evidence of a pre-existing T cell immunity against the pathogen-derived epitopes, matching to T cell response against the paired TPNAs. Such effect was more evident for the vaccinia virus epitope and its homologous TPNA PDCD7_mut, suggesting a role for the immunological memory established upon the pediatric vaccination. The data observed in this patient strongly suggest a relevant role for a pre-existing T cell immunity specific against a pathogen derived epitope and better control of tumor progression. Additional studies on larger number of samples are currently on going for validation. These findings suggest that the same in silico strategy can be used to identify tumor associated antigens (TAAs) with homology to pathogen-derived epitopes, which could elicit a cross-reacting immune response with a more potent anti-tumor effect. Moreover, it is also conceivable to modify TAAs in order to generate heteroclitic epitopes sharing homologies with pathogen-derived antigens. Such peptides should be able to elicit an effective cross-reactive anti-tumor immune response [34, 35].

In addition, the long term survival of the HLA-008 patient correlated also with a favorable intra-tumor immune pattern (so called “immunophenogram”), characterized by high CD8+ T cell and low Tregs infiltration, low expression of immune checkpoint molecules and high expression of HLA molecules.

**Fig. 6:**
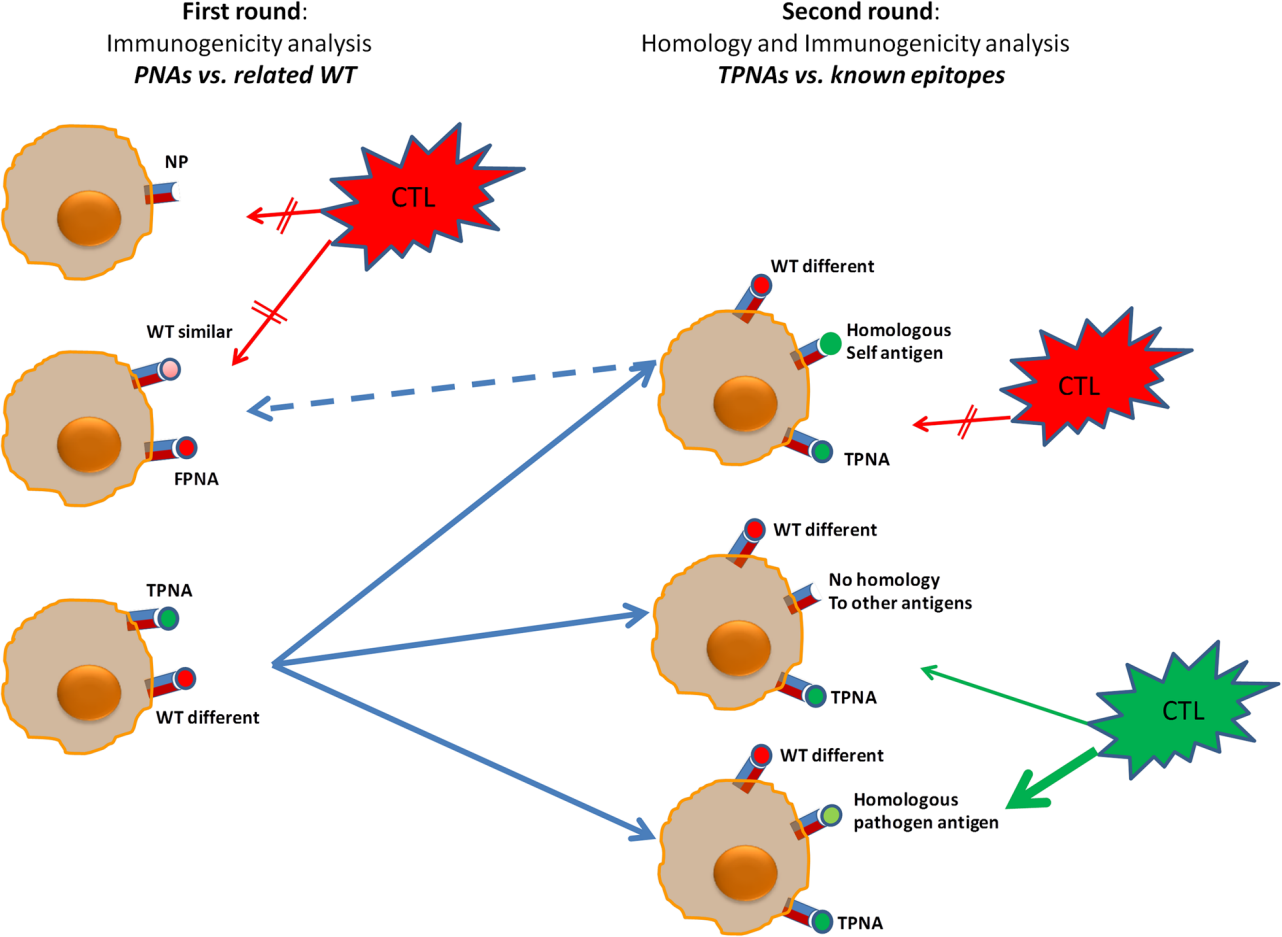
Pipeline for TPNAs discovery. Discovery of TPNAs is based on a two-round bioinformatics process. In the first round, TPNAs are identified to be different from related epitopes. In the second round, TPNAs are confirmed if no homology is found with any known epitope or if homology is found with pathogen-related epitopes. In the latter case, TPNAs are more efficient target of pre-existing T cell memory

The results described in the present study suggest that a very limited fraction of mutations are immunologically relevant, providing the tumor an enormous advantage to grow undisturbed. However, we provide a strong evidence that a simple prediction for immunogenicity is not sufficient for identifying a neoantigen. Indeed, a true predicted neoantigen (TPNA) should (1) derive from a wild type peptide which is not immunogenic; and, (2) lack any homology with any other wild type self epitope. In addition, if TPNAs show homology with pathogen-derived epitopes, the pre-existing pathogen-specific immunity will respond faster and stronger to TPNAs, resulting in a more efficient control of the tumor evolution and, consequently, in a better clinical outcome, as observed in the patient HLA-008 (Fig. 6). Indeed, overall, our results clearly suggest that the quality of TPNAs more than the quantity correlates with patients’ survival.

In general, we believe that the application of the described algorithm for neoantigen discovery will provide the real tumor-specific targets for immunotherapies (e.g. therapeutic vaccines, adoptive T cell therapies) and will help to shed the light on the mechanisms of the reported poor responsiveness in treatments with checkpoint inhibitors.

## Conclusions

The main finding of this study is that predicted neoantigens may not be “new antigens”. Indeed, several of such predicted neoantigens are not distinguishable from the corresponding wild type epitopes and/or share homology with other human cellular self antigens. Cancer cells carrying such “false” neoantigens are immunologically “invisible” and may grow undisturbed. Consequently, the “false” neoantigens are useless for cancer immunotherapy strategies.

“True” neoantigens are only those which don’t share any homology with any human self antigen or, even better, which share homology with infectious disease-derived epitopes. In the latter case, a pre-existing T cell memory may respond better and swifter, resulting in either long-term survival or responsiveness to checkpoint inhibitors. Consequently, the “true” neoantigens are the only antigens which may be effective in cancer immunotherapy strategies.

Overall, we show that the quality more than the quantity of neoantigens expressed by cancer cells may predict clinical outcome as well as guide selection of the most appropriate target antigens for cancer immunotherapy in HCC patients.

## Additional files

**Additional file 1.** Additional tables.

**Additional file 2: Fig. S1.** **(A)** Principal component analysis performed on normalized gene expression values showing for each patient a clear segregation between tumor and the adjacent liver tissue. **Fig. S2.** KEGG signaling pathway analysis of relevant oncogenes **(A**–**D)**. In each pathway, genes modulated in HCC samples are indicated in red. **Fig. S3. (A)** Flow chart of selection of mutations for neoantigen discovery. **(B)** Number of mutations identified for each HCC sample. **(C)** Percentage of mutations with different biological impact on cellular functions. **Fig. S4.** Venn diagrams showing the number of common and unique PNAs, predicted by the NetTepi and NetMHCstabpan servers for each sample. **Fig. S5.** Number of common PNAs, TPNAs and FPNAs predicted by both the NetTepi and NetMHCstabpan servers for each sample. **Fig. S6.** Venn diagrams showing the number of common and unique TPNAs, predicted by the NetTepi and NetMHCstabpan servers for each sample. **Fig. S7.** Homology analysis of TCR binding amino acid residues between FPNAs identified in each HCC sample and the corresponding wild type peptides. Green amino acids indicate homology between FPNA and wild type; mismatch is represented by indicating both amino acids found at that position. **Fig. S8.** Alignment of TPNAs amino acid sequences. Amino acid sequences of all TPNAs or divided into low or high %Ranks were aligned to generate a sequence logo. The height of the stack indicates the sequence conservation at that position, while the height of symbols within the stack indicates the relative frequency of each amino or nucleic acid at that position. Alignment of the entire epitopes (A–C) or of the four TCR binding residues (D, F). **Fig. S9.** Homology analysis of TCR binding amino acid residues between TPNAs identified in each HCC sample and the indicated homologous human or infectious disease-related epitopes. Green amino acids indicate homology between the sequences; full mismatch is represented by red amino acids; mismatch amino acids of the same class is represented by orange amino acids.

## Abbreviations

TPNAs: true predicted neoantigens
HCV: hepatitis C virus
HCC: hepatocellular carcinoma
TILs: tumor infiltrating lymphocytes
HBV: hepatitis B virus
PBMCs: peripheral blood mononuclear cells
PCA: principal component analysis
DEGs: differentially expressed genes
FDR: false discovery rate
SNP: single nucleotide polymorphism
SNVs: single nucleotide variations
BFA: brefeldin A
MFI: mean fluorescence index
IFN-γ: interferon gamma
PBS: phosphate-buffered saline
PHA: phytohemagglutinin
NP: not predicted neoantigens
PNAs: predicted neoantigens
FPNAs: false predicted neoantigens
TCR: T cell receptor
TAAs: tumor associated antigens

## References

[1] Greenman C, Stephens P, Smith R, Dalgliesh GL, Hunter C, Bignell G (2007). Patterns of somatic mutation in human cancer genomes. Nature.

[2] Stratton MR (2011). Exploring the genomes of cancer cells: progress and promise. Science.

[3] Schumacher TN, Schreiber RD (2015). Neoantigens in cancer immunotherapy. Science.

[4] Yarchoan M, Johnson BA, Lutz ER, Laheru DA, Jaffee EM (2017). Targeting neoantigens to augment antitumour immunity. Nat Rev Cancer.

[5] Lennerz V, Fatho M, Gentilini C, Frye RA, Lifke A, Ferel D (2005). The response of autologous T cells to a human melanoma is dominated by mutated neoantigens. Proc Natl Acad Sci USA.

[6] Brown SD, Warren RL, Gibb EA, Martin SD, Spinelli JJ, Nelson BH (2014). Neo-antigens predicted by tumor genome meta-analysis correlate with increased patient survival. Genome Res.

[7] Lu YC, Yao X, Crystal JS, Li YF, El-Gamil M, Gross C (2014). Efficient identification of mutated cancer antigens recognized by T cells associated with durable tumor regressions. Clin Cancer Res.

[8] Tran E, Turcotte S, Gros A, Robbins PF, Lu YC, Dudley ME (2014). Cancer immunotherapy based on mutation-specific CD4+ T cells in a patient with epithelial cancer. Science.

[9] Li F, Chen C, Ju T, Gao J, Yan J, Wang P (2016). Rapid tumor regression in an Asian lung cancer patient following personalized neo-epitope peptide vaccination. Oncoimmunology..

[10] Sahin U, Derhovanessian E, Miller M, Kloke BP, Simon P, Lower M (2017). Personalized RNA mutanome vaccines mobilize poly-specific therapeutic immunity against cancer. Nature.

[11] Ott PA, Hu Z, Keskin DB, Shukla SA, Sun J, Bozym DJ (2017). An immunogenic personal neoantigen vaccine for patients with melanoma. Nature.

[12] Melief CJ, van der Burg SH (2008). Immunotherapy of established (pre)malignant disease by synthetic long peptide vaccines. Nat Rev Cancer.

[13] Hamanishi J, Mandai M, Matsumura N, Abiko K, Baba T, Konishi I (2016). PD-1/PD-L1 blockade in cancer treatment: perspectives and issues. Int J Clin Oncol..

[14] Rizvi NA, Hellmann MD, Snyder A, Kvistborg P, Makarov V, Havel JJ (2015). Cancer immunology. Mutational landscape determines sensitivity to PD-1 blockade in non-small cell lung cancer. Science..

[15] Snyder A, Makarov V, Merghoub T, Yuan J, Zaretsky JM, Desrichard A (2014). Genetic basis for clinical response to CTLA-4 blockade in melanoma. N Engl J Med..

[16] Luksza M, Riaz N, Makarov V, Balachandran VP, Hellmann MD, Solovyov A (2017). A neoantigen fitness model predicts tumour response to checkpoint blockade immunotherapy. Nature.

[17] Ghorani E, Rosenthal R, McGranahan N, Reading JL, Lynch M, Peggs KS (2018). Differential binding affinity of mutated peptides for MHC class I is a predictor of survival in advanced lung cancer and melanoma. Ann Oncol.

[18] Rech AJ, Balli D, Mantero A, Ishwaran H, Nathanson KL, Stanger BZ (2018). Tumor immunity and survival as a function of alternative neopeptides in human cancer. Cancer Immunol Res..

[19] Balachandran VP, Luksza M, Zhao JN, Makarov V, Moral JA, Remark R (2017). Identification of unique neoantigen qualities in long-term survivors of pancreatic cancer. Nature.

[20] Buonaguro L, Petrizzo A, Tagliamonte M, Tornesello ML, Buonaguro FM (2013). Challenges in cancer vaccine development for hepatocellular carcinoma. J Hepatol.

[21] Buonaguro L, HEPAVAC Consortium (2016). Developments in cancer vaccines for hepatocellular carcinoma. Cancer Immunol Immunother.

[22] Tagliamonte M, Petrizzo A, Tornesello ML, Ciliberto G, Buonaguro FM, Buonaguro L (2016). Combinatorial immunotherapy strategies for hepatocellular carcinoma. Curr Opin Immunol.

[23] Fujimoto A, Furuta M, Totoki Y, Tsunoda T, Kato M, Shiraishi Y (2016). Whole-genome mutational landscape and characterization of noncoding and structural mutations in liver cancer. Nat Genet.

[24] Li S, Mao M (2013). Next generation sequencing reveals genetic landscape of hepatocellular carcinomas. Cancer Lett.

[25] Schulze K, Imbeaud S, Letouze E, Alexandrov LB, Calderaro J, Rebouissou S (2015). Exome sequencing of hepatocellular carcinomas identifies new mutational signatures and potential therapeutic targets. Nat Genet.

[26] Totoki Y, Tatsuno K, Yamamoto S, Arai Y, Hosoda F, Ishikawa S (2011). High-resolution characterization of a hepatocellular carcinoma genome. Nat Genet.

[27] Costa V, Esposito R, Ziviello C, Sepe R, Bim LV, Cacciola NA (2015). New somatic mutations and WNK1-B4GALNT3 gene fusion in papillary thyroid carcinoma. Oncotarget..

[28] Kim D, Pertea G, Trapnell C, Pimentel H, Kelley R, Salzberg SL (2013). TopHat2: accurate alignment of transcriptomes in the presence of insertions, deletions and gene fusions. Genome Biol..

[29] Russo F, Angelini C (2014). RNASeqGUI: a GUI for analysing RNA-Seq data. Bioinformatics.

[30] Binkowski TA, Marino SR, Joachimiak A (2012). Predicting HLA class I non-permissive amino acid residues substitutions. PLoS ONE..

[31] Charoentong P, Finotello F, Angelova M, Mayer C, Efremova M, Rieder D (2017). Pan-cancer immunogenomic analyses reveal genotype-immunophenotype relationships and predictors of response to checkpoint blockade. Cell Rep..

[32] Efremova M, Finotello F, Rieder D, Trajanoski Z (2017). Neoantigens generated by individual mutations and their role in cancer immunity and immunotherapy. Front Immunol..

[33] Duan F, Duitama J, Al SS, Ayres CM, Corcelli SA, Pawashe AP (2014). Genomic and bioinformatic profiling of mutational neoepitopes reveals new rules to predict anticancer immunogenicity. J Exp Med.

[34] Loftus DJ, Castelli C, Clay TM, Squarcina P, Marincola FM, Nishimura MI (1996). Identification of epitope mimics recognized by CTL reactive to the melanoma/melanocyte-derived peptide MART-1(27–35). J Exp Med.

[35] Vujanovic L, Shi J, Kirkwood JM, Storkus WJ, Butterfield LH (2014). Molecular mimicry of MAGE-A6 and Mycoplasma penetrans HF-2 epitopes in the induction of antitumor CD8(+) T-cell responses. Oncoimmunology..

