# Supplementary material for: Unique true predicted neoantigens (TPNAs) correlates with anti-tumor immune control in HCC patients
Source: J Transl Med. 2018 Oct 19;16:286. doi: 10.1186/s12967-018-1662-9 (PMC6194606; doi:10.1186/s12967-018-1662-9)
Supplement: Supplementary file 1 — Additional file 1. Additional tables. [file 12967_2018_1662_MOESM1_ESM.docx]

**Table S1.** Characteristics of HCC patients enrolled in the study

| Code Nr. | Sex | Age | HLA-A *0201 | HLA-A *2402 | Date of surgery | T.N.M. | Grading | Disease evolution |
| --- | --- | --- | --- | --- | --- | --- | --- | --- |
| HLA-008 | F | 77 | POS | POS | 29/03/2013 | T2 N0 M0 | G2 | Alive |
| HLA-009 | M | 77 | POS | NEG | 05/04/2013 | T2 N0 M0 | G2 | Alive |
| HLA-012 | F | 62 | POS | NEG | 10/05/2013 | T3 N0 M0 | G2 | Alive |
| HLA-016 | M | 69 | POS | NEG | 12/06/2013 | T1 N0 M0 | G2 | Deceased |
| HLA-017 | M | 75 | POS | NEG | 18/06/2013 | T3 N0 M1 | G2 | Deceased |
| HLA-022 | M | 74 | POS | NEG | 09/10/2013 | T3 N0 M0 | G2 | Deceased |
| HLA-026 | M | 80 | POS | POS | 22/01/2014 | T2 N0 M0 | G2 | Deceased |
| HLA-028 | M | 68 | POS | NEG | 09/04/2014 | T3 N0 M0 | G2 | Deceased |
| HLA-029 | M | 74 | POS | NEG | 18/04/2014 | T3 N0 M0 | G2 | Deceased |

**Table S2.** Common genes bearing unique non-synonymous SNVs.


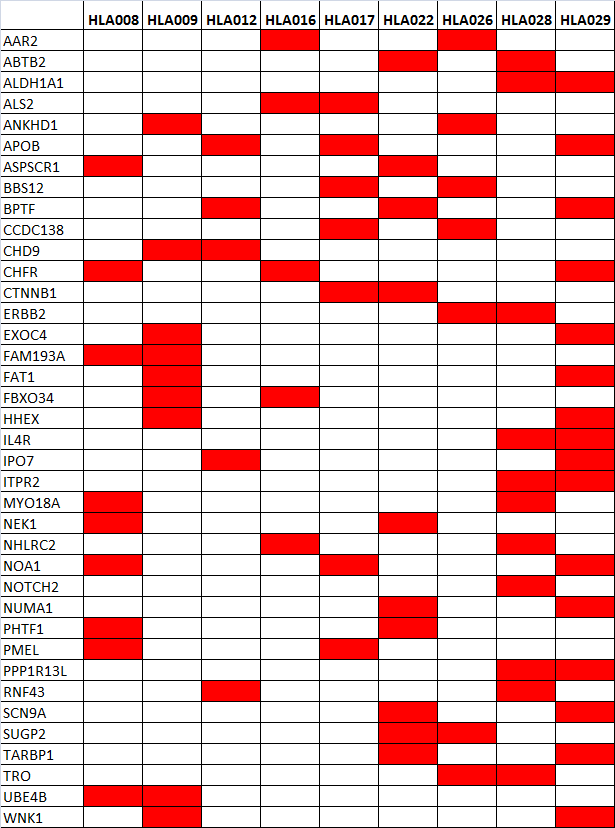


**Table S3.** Epitope prediction analysis per each HCC sample by NetTepi algorithm (%Rank ≤2). NP antigens (white); FPNAs (orange); TPNAs (green).

**Table S4.** Predicted TPNAs with high prediction rank.

**Table S5.** Sequence homology between TPNAs and known human epitopes.

**Table S6.** Sequence homology between TPNAs and known infectious disease-derived epitopes.

**Table S7**. Binding levels of peptides used in the mouse C57BL/6 model.
