# Supplementary material for: Unique true predicted neoantigens (TPNAs) correlates with anti-tumor immune control in HCC patients
Source: J Transl Med. 2018 Oct 19;16:286. doi: 10.1186/s12967-018-1662-9 (PMC6194606; doi:10.1186/s12967-018-1662-9)
Supplement: Supplementary file 2 — Additional file 2: Fig. S1. (A) Principal component analysis performed on normalized gene expression values showing for each patient a clear segregation between tumor and the adjacent liver tissue. Fig. S2. KEGG signaling pathway analysis of relevant oncogenes (A–D). In each pathway, genes modulated in HCC samples are indicated in red. Fig. S3. (A) Flow chart of selection of mutations for neoantigen discovery. (B) Number of mutations identified for each HCC sample. (C) Percentage of mutations with different biological impact on cellular functions. Fig. S4. Venn diagrams showing the number of common and unique PNAs, predicted by the NetTepi and NetMHCstabpan servers for each sample. Fig. S5. Number of common PNAs, TPNAs and FPNAs predicted by both the NetTepi and NetMHCstabpan servers for each sample. Fig. S6. Venn diagrams showing the number of common and unique TPNAs, predicted by the NetTepi and NetMHCstabpan servers for each sample. Fig. S7. Homology analysis of TCR binding amino acid residues between FPNAs identified in each HCC sample and the corresponding wild type peptides. Green amino acids indicate homology between FPNA and wild type; mismatch is represented by indicating both amino acids found at that position. Fig. S8. Alignment of TPNAs amino acid sequences. Amino acid sequences of all TPNAs or divided into low or high %Ranks were aligned to generate a sequence logo. The height of the stack indicates the sequence conservation at that position, while the height of symbols within the stack indicates the relative frequency of each amino or nucleic acid at that position. Alignment of the entire epitopes (A–C) or of the four TCR binding residues (D, F). Fig. S9. Homology analysis of TCR binding amino acid residues between TPNAs identified in each HCC sample and the indicated homologous human or infectious disease-related epitopes. Green amino acids indicate homology between the sequences; full mismatch is represented by red amino acids; mismatch amino acids o [file 12967_2018_1662_MOESM2_ESM.pptx]

## Slide 1
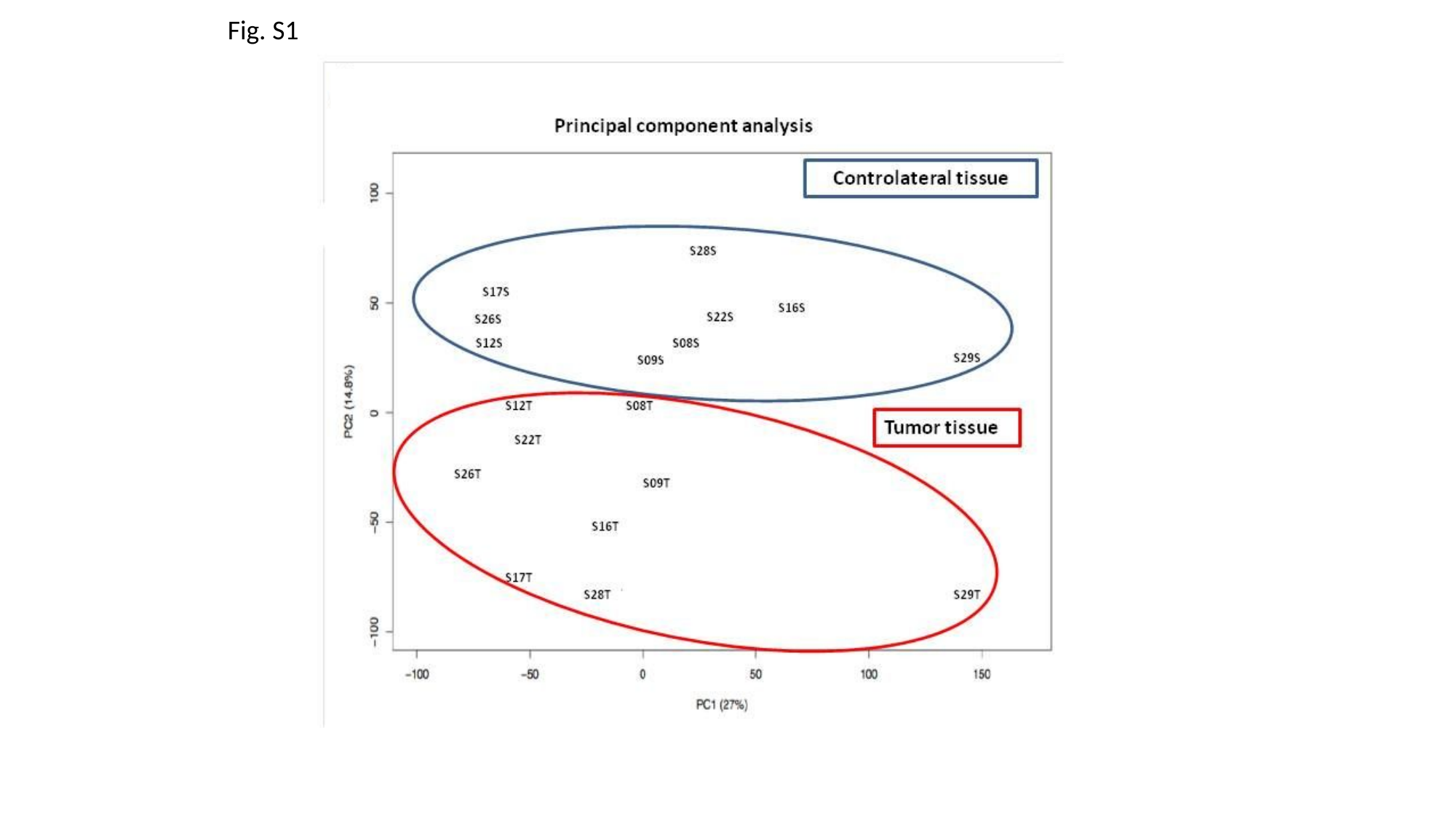

Fig. S1

## Slide 2
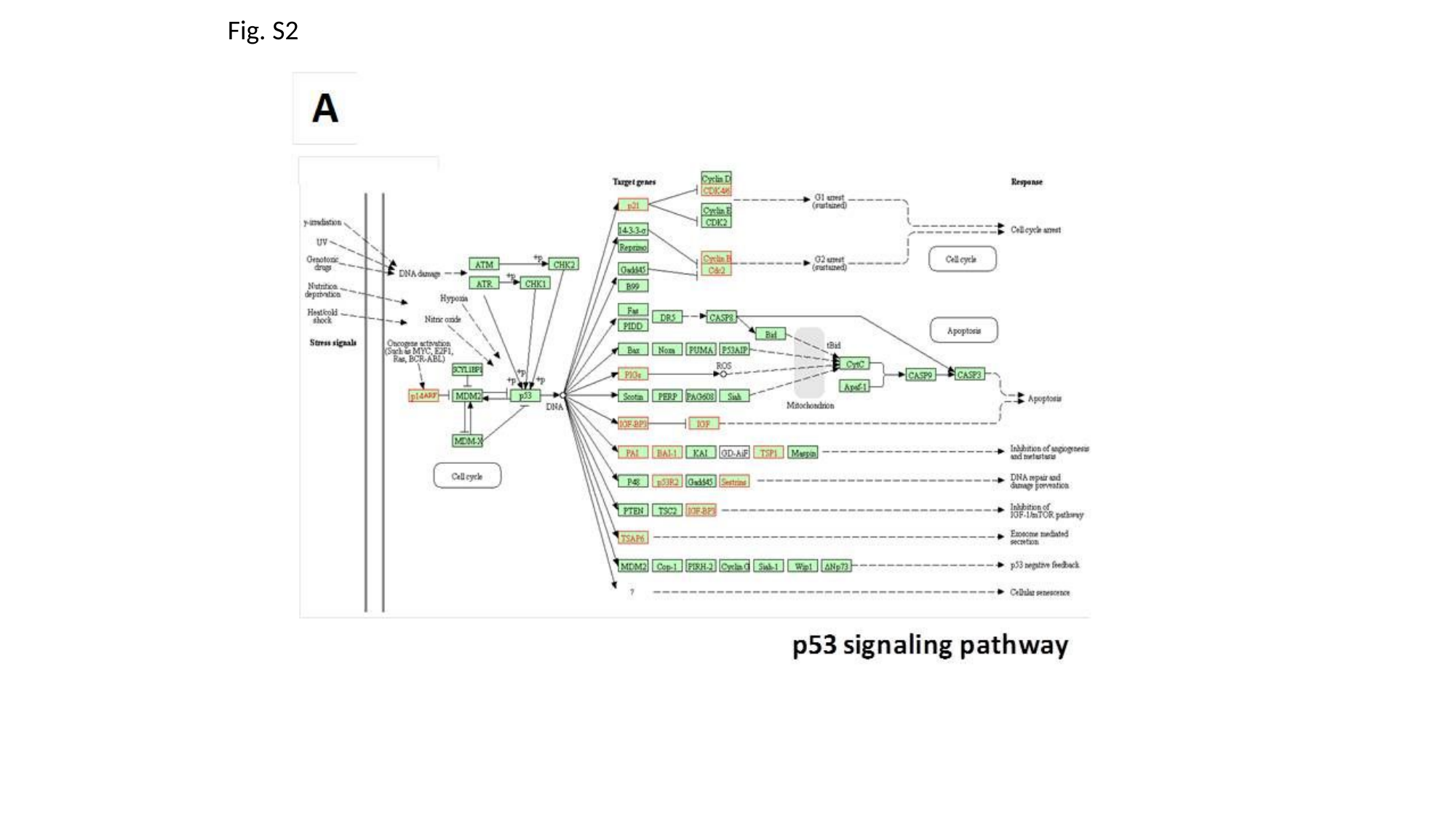

Fig. S2

## Slide 3
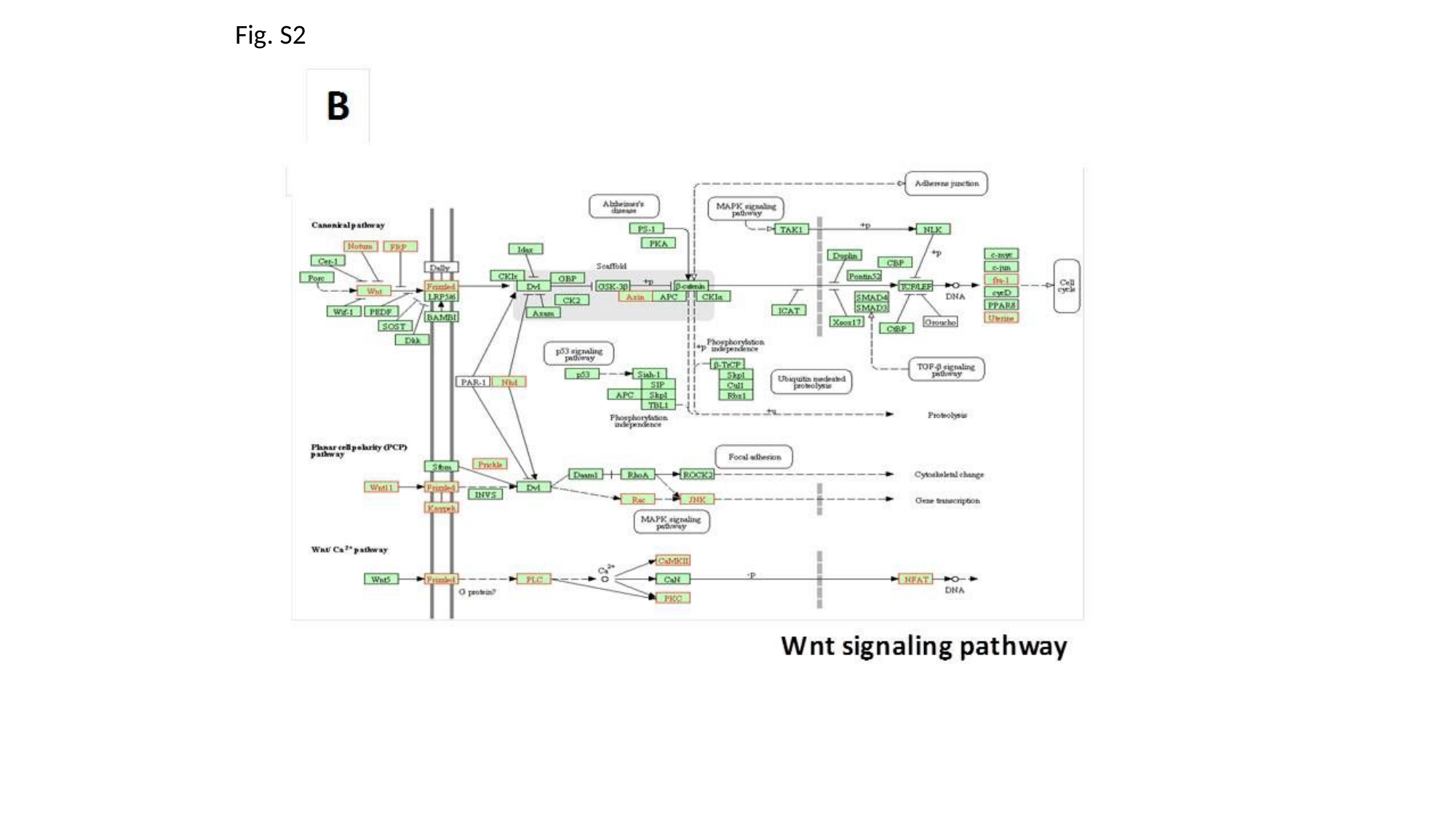

Fig. S1
Fig. S2

## Slide 4
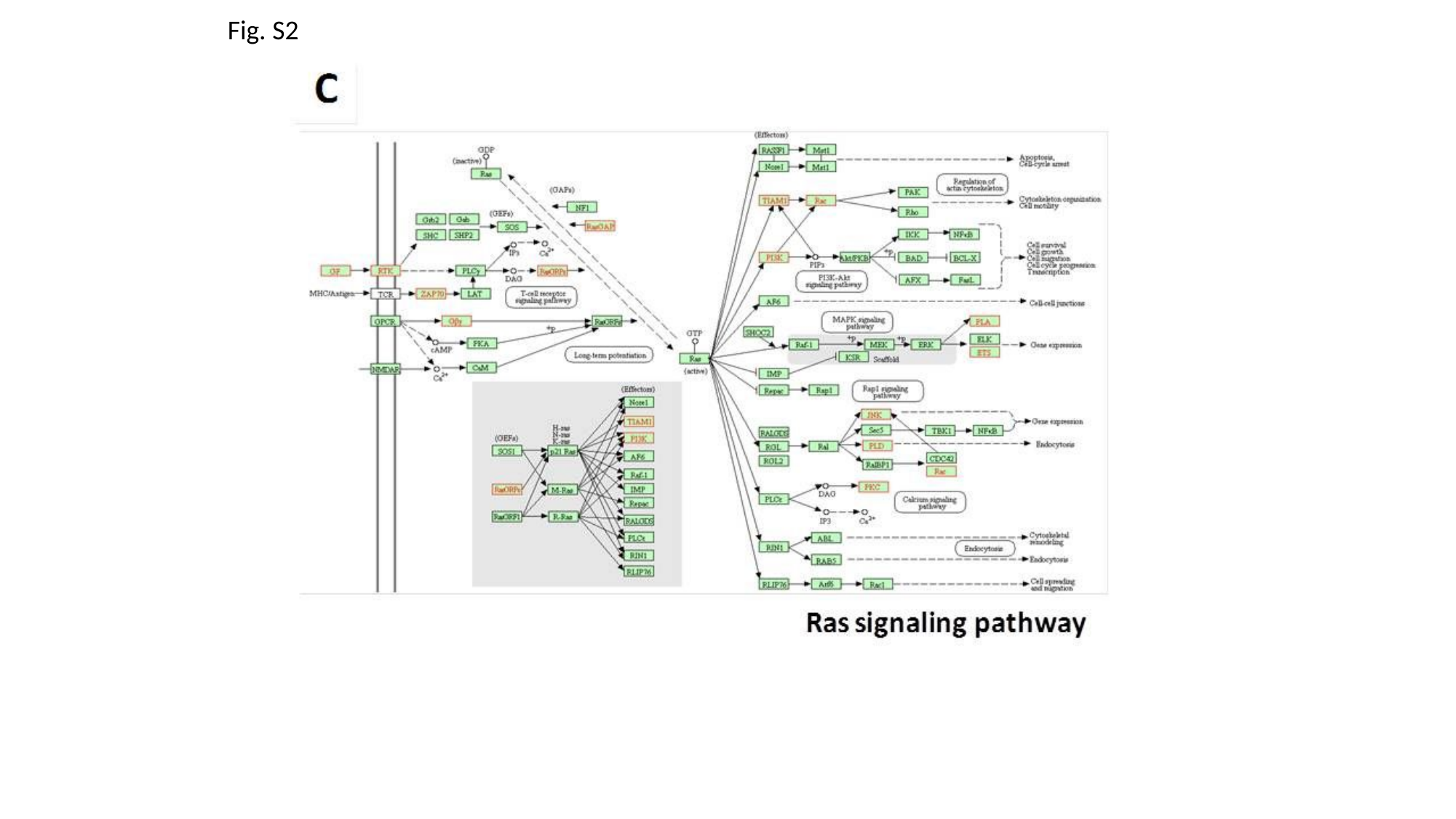

Fig. S2

## Slide 5
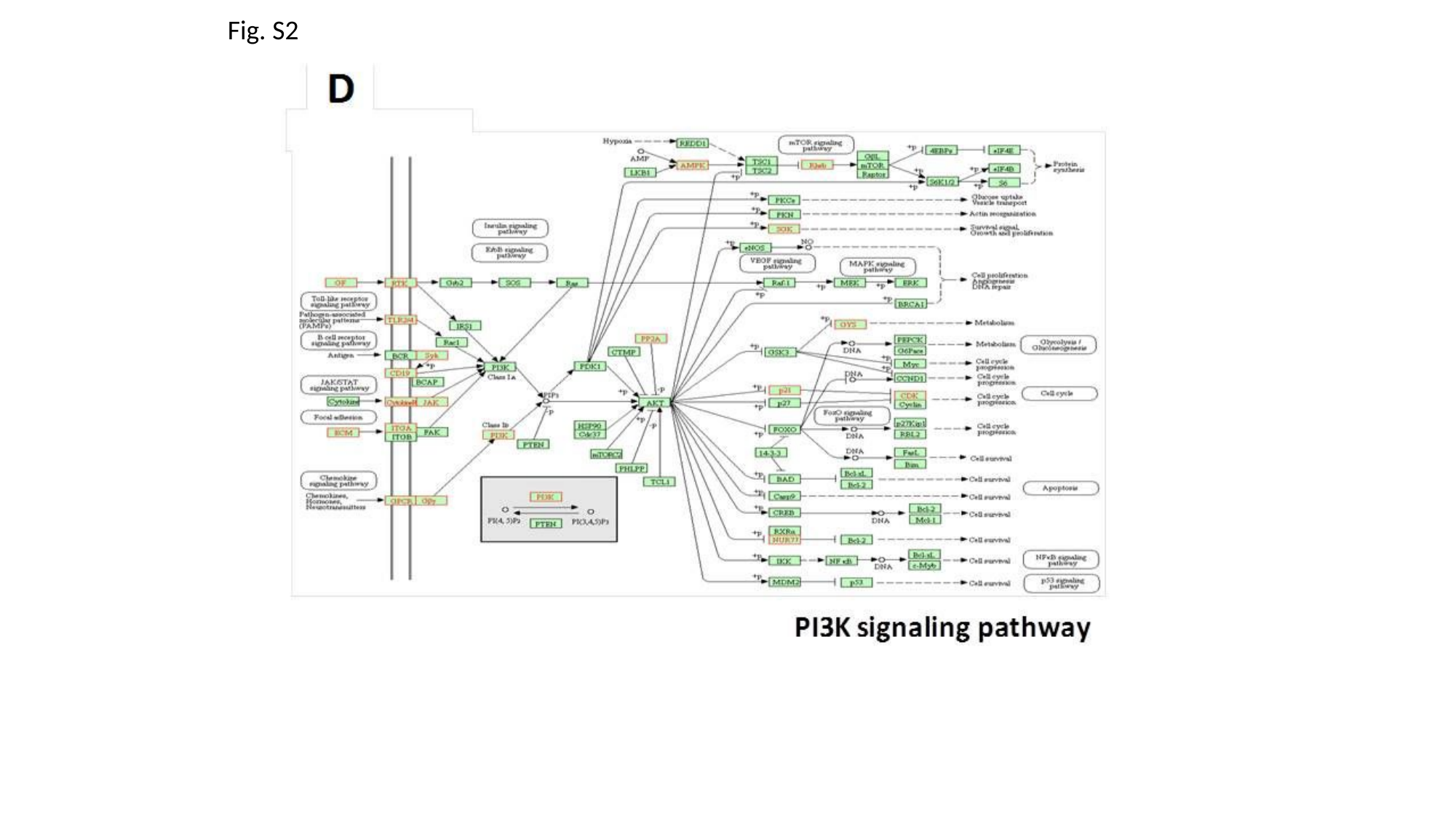

Fig. S2

## Slide 6
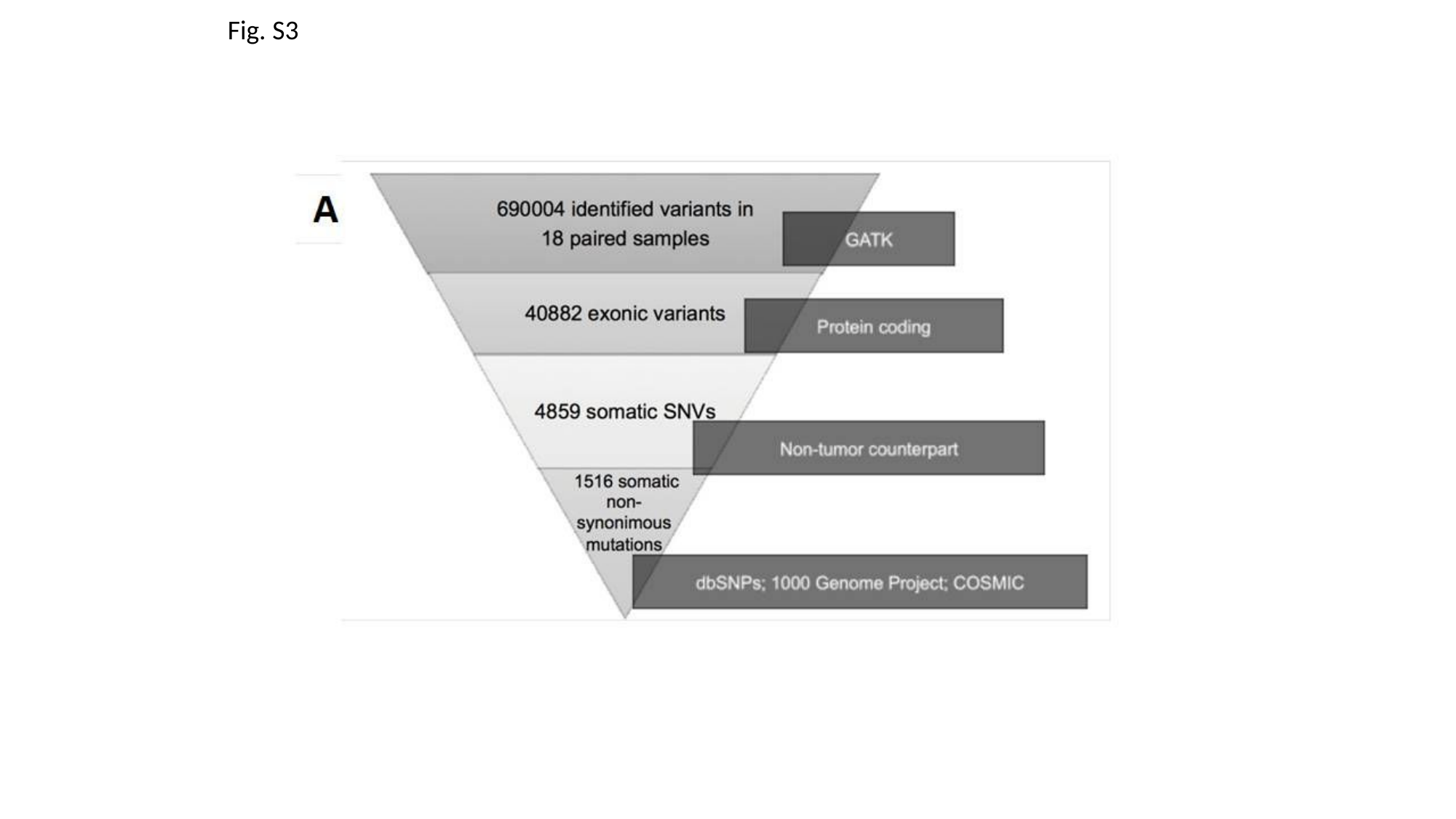

Fig. S3

## Slide 7
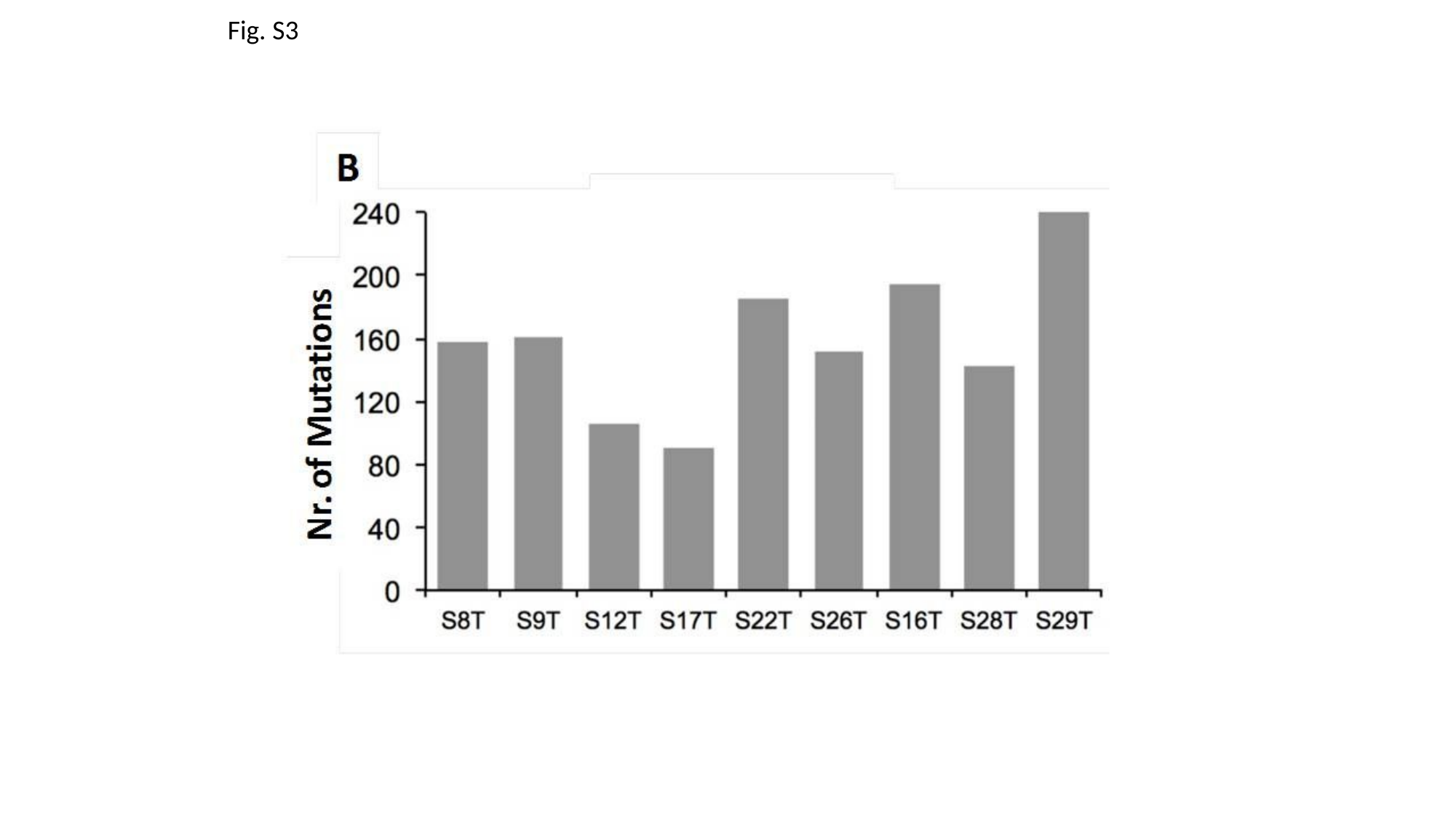

Fig. S3

## Slide 8
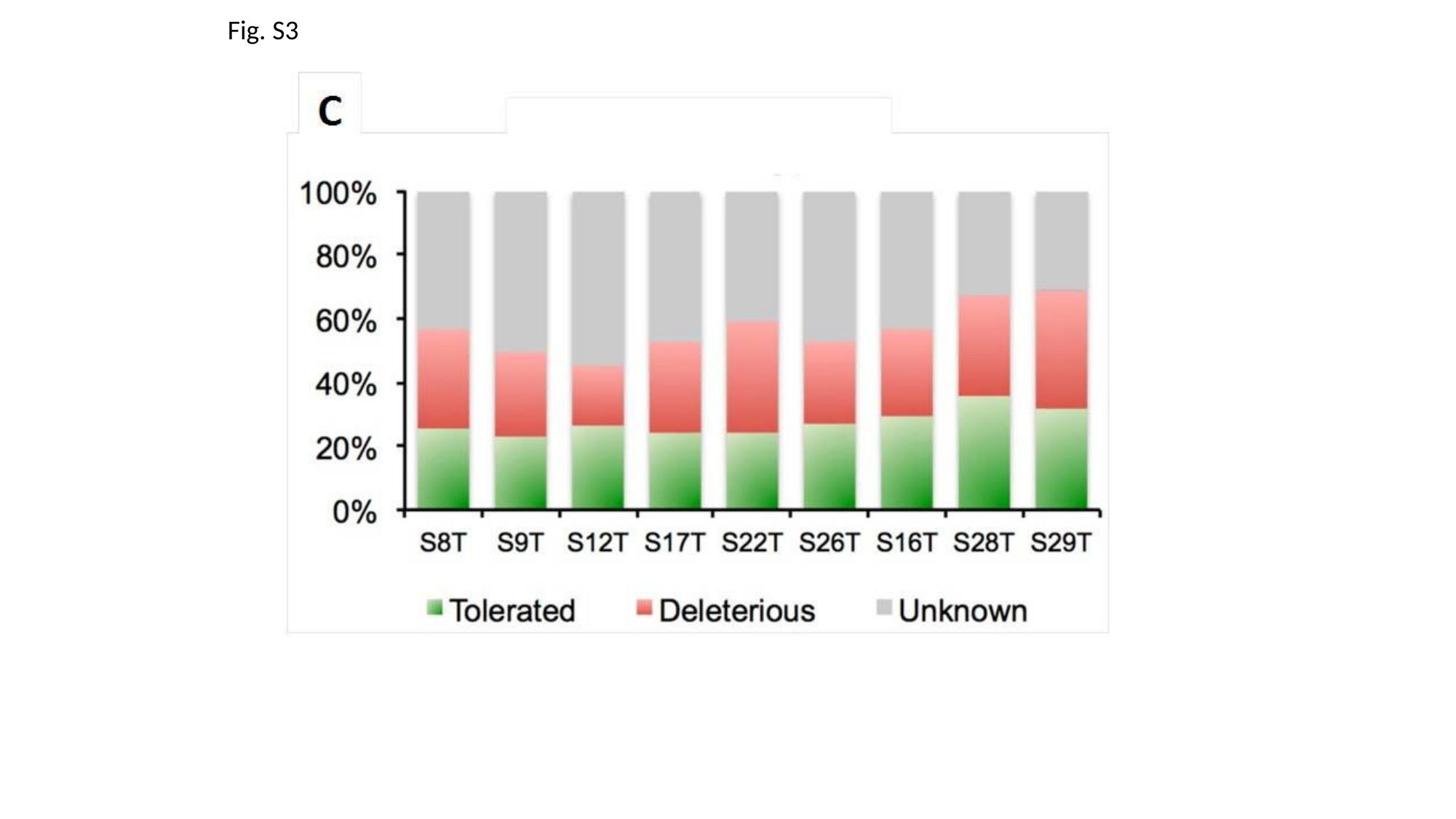

Fig. S3

## Slide 9
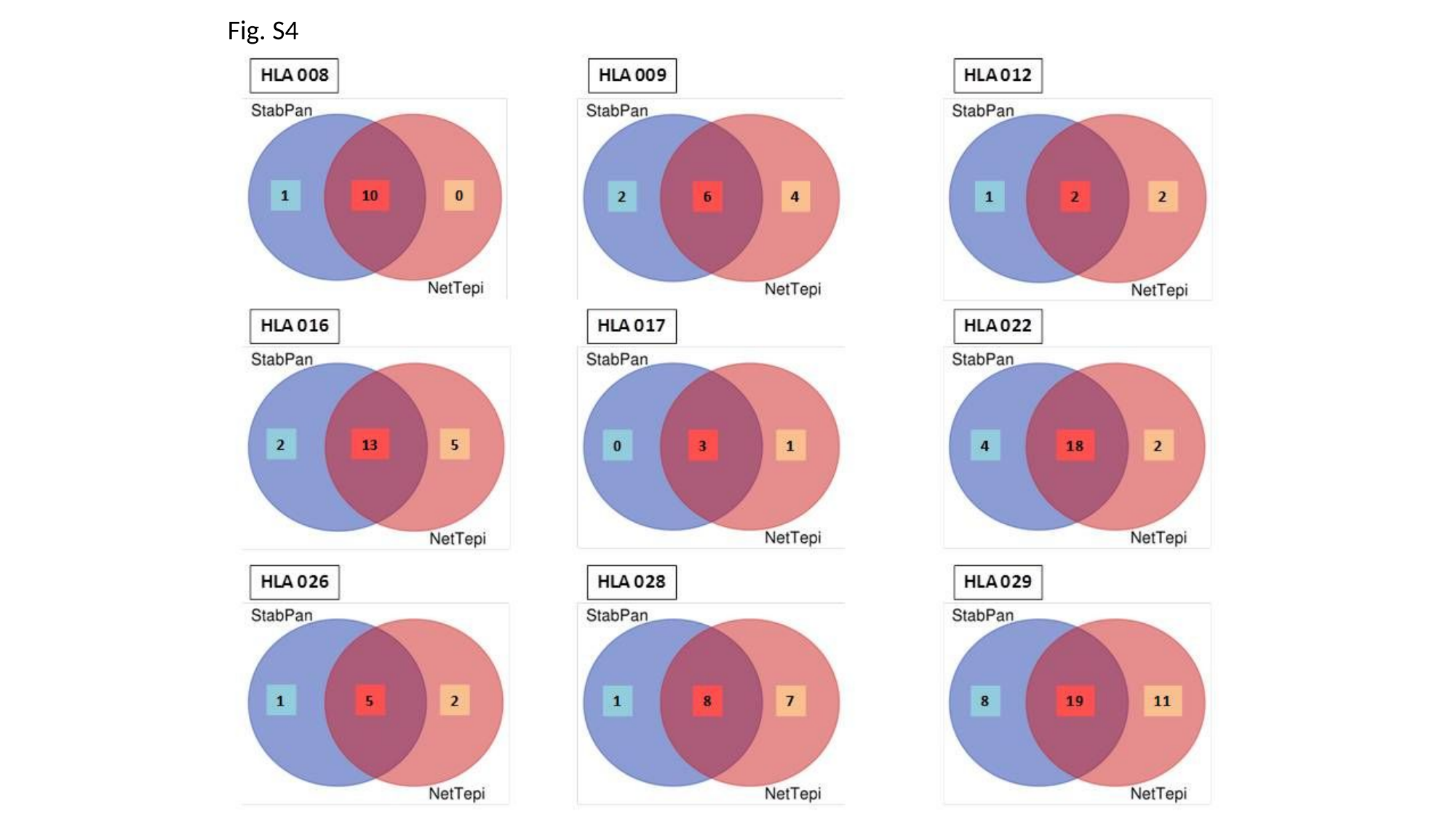

Fig. S4

## Slide 10
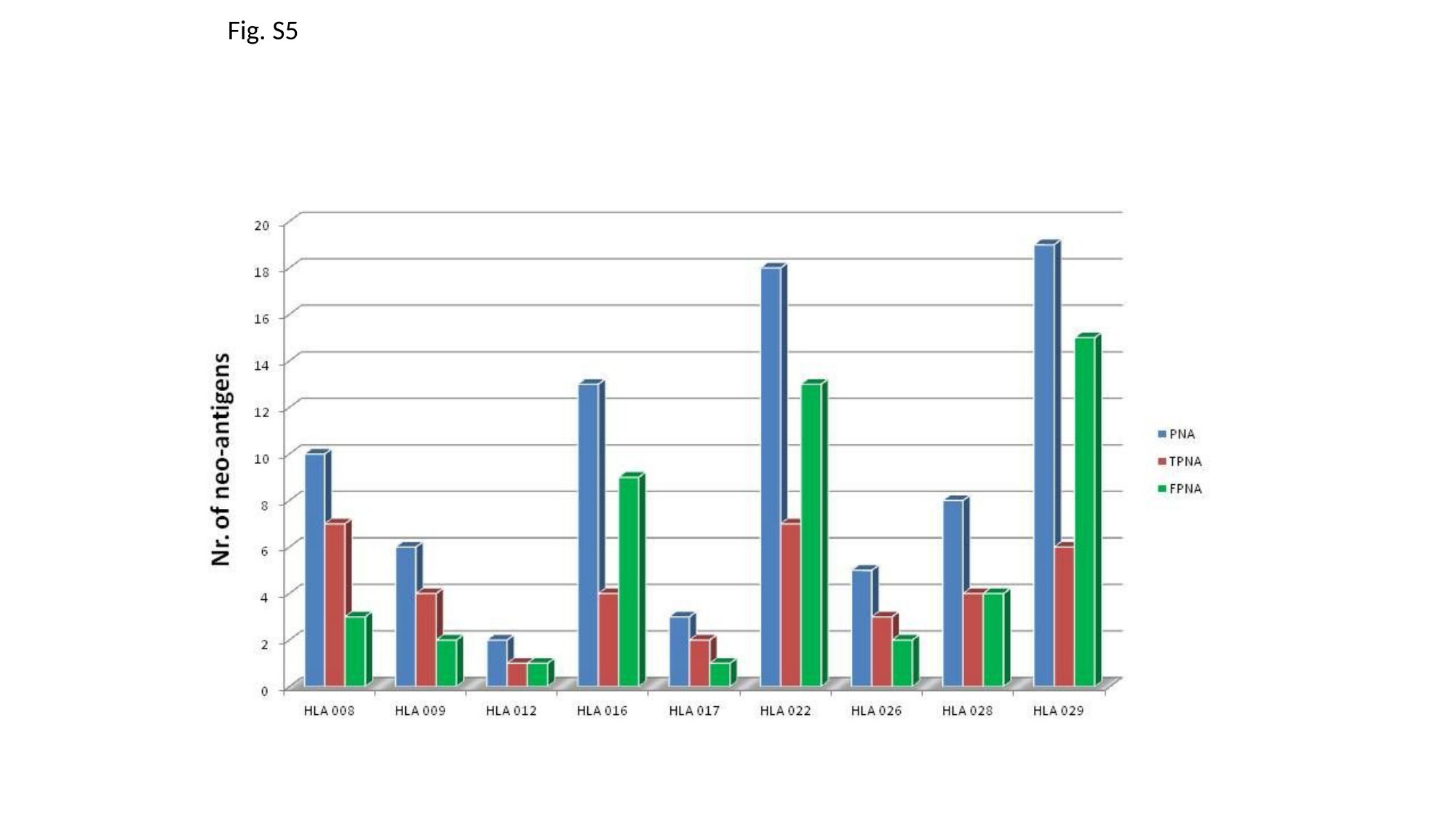

Fig. S5

## Slide 11
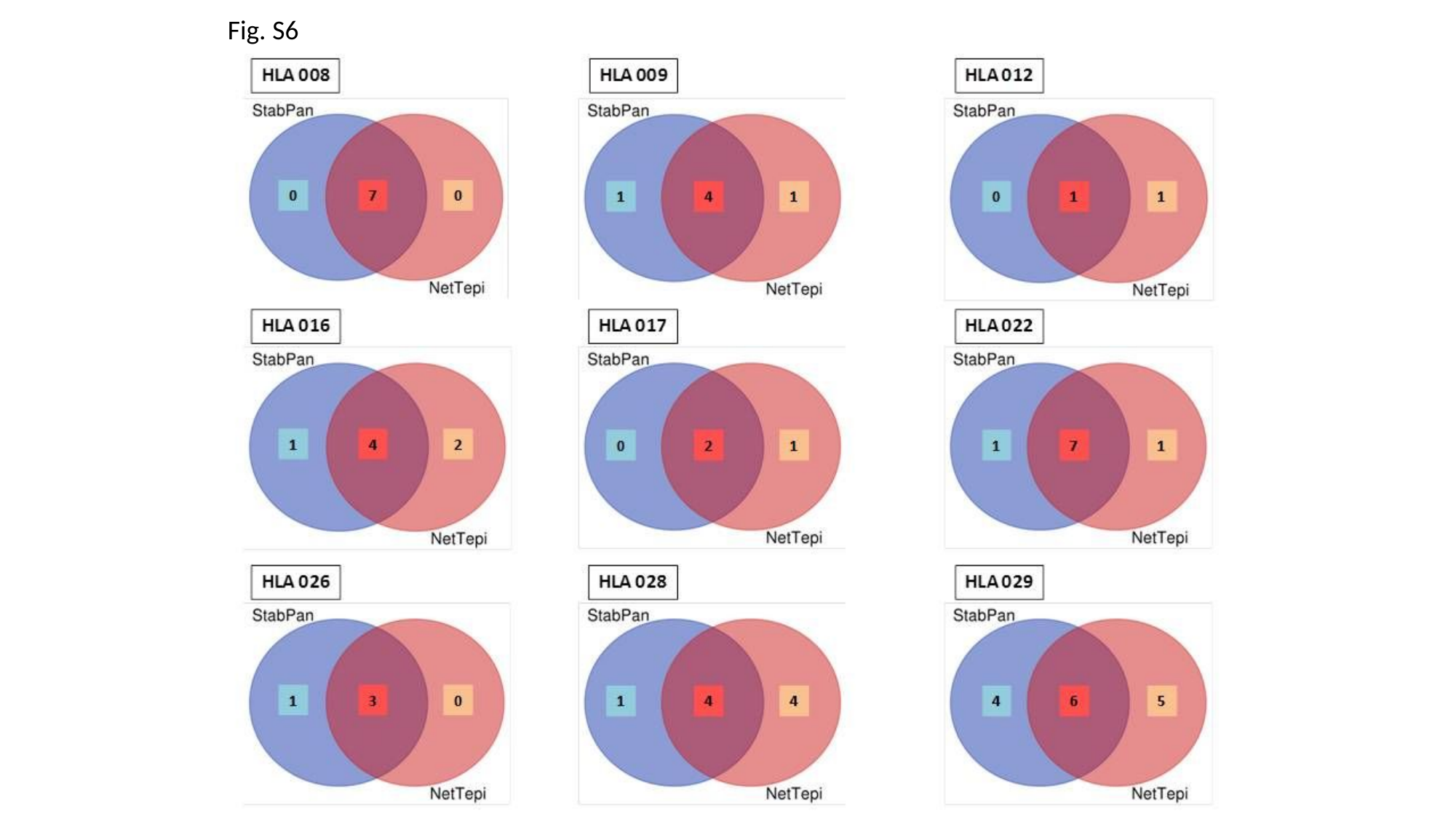

Fig. S6

## Slide 12
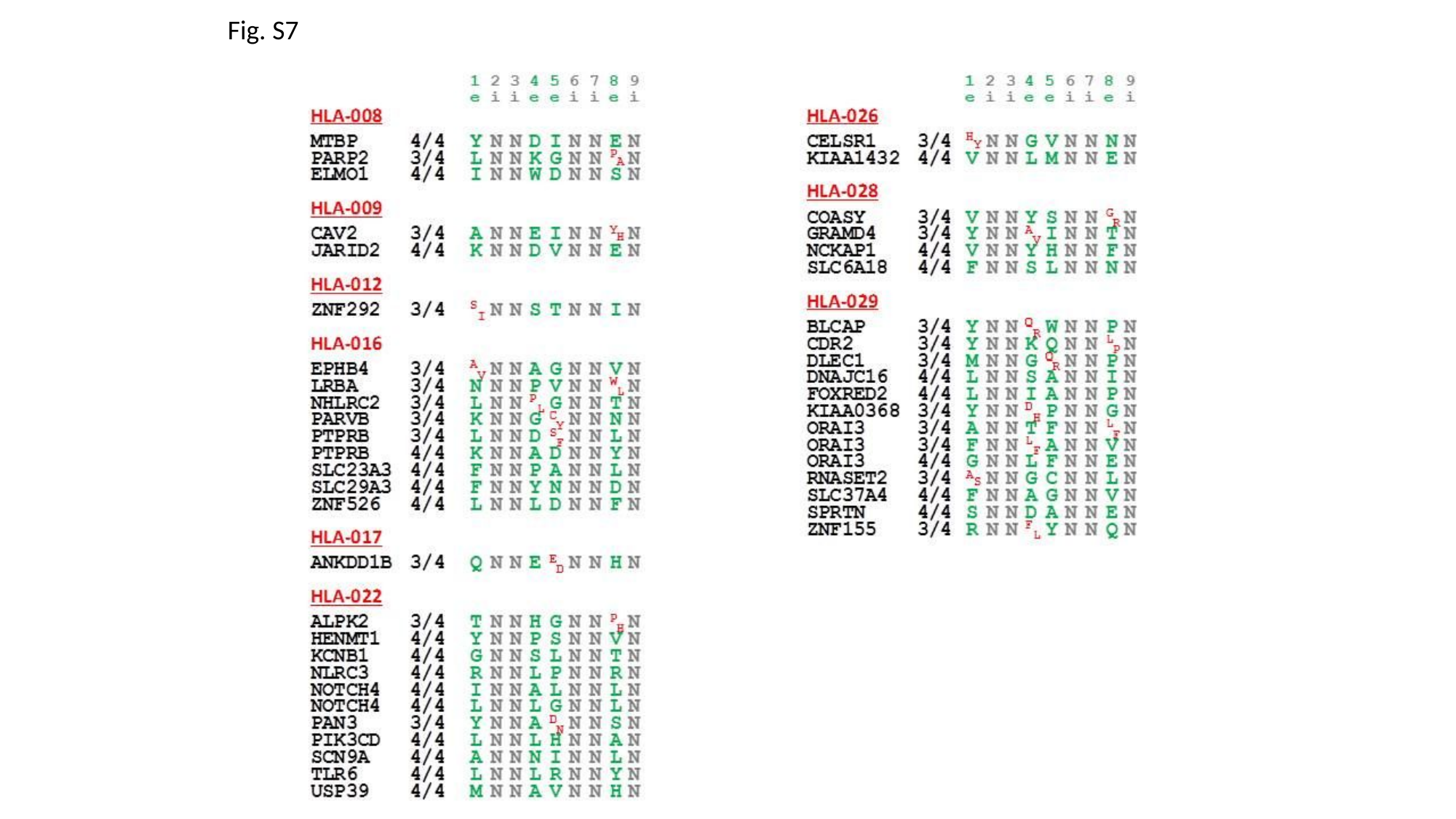

Fig. S7

## Slide 13
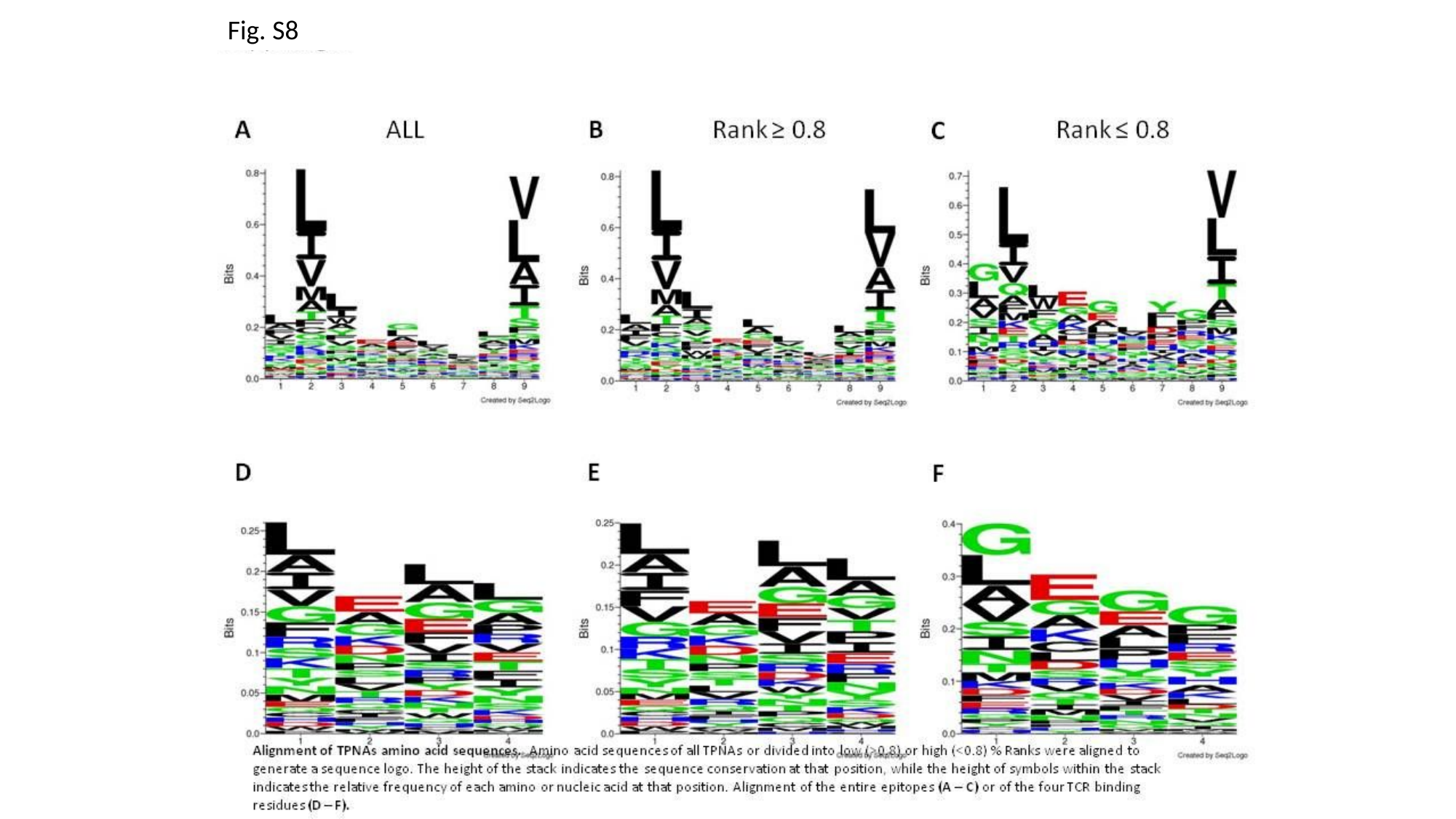

Fig. S8

## Slide 14
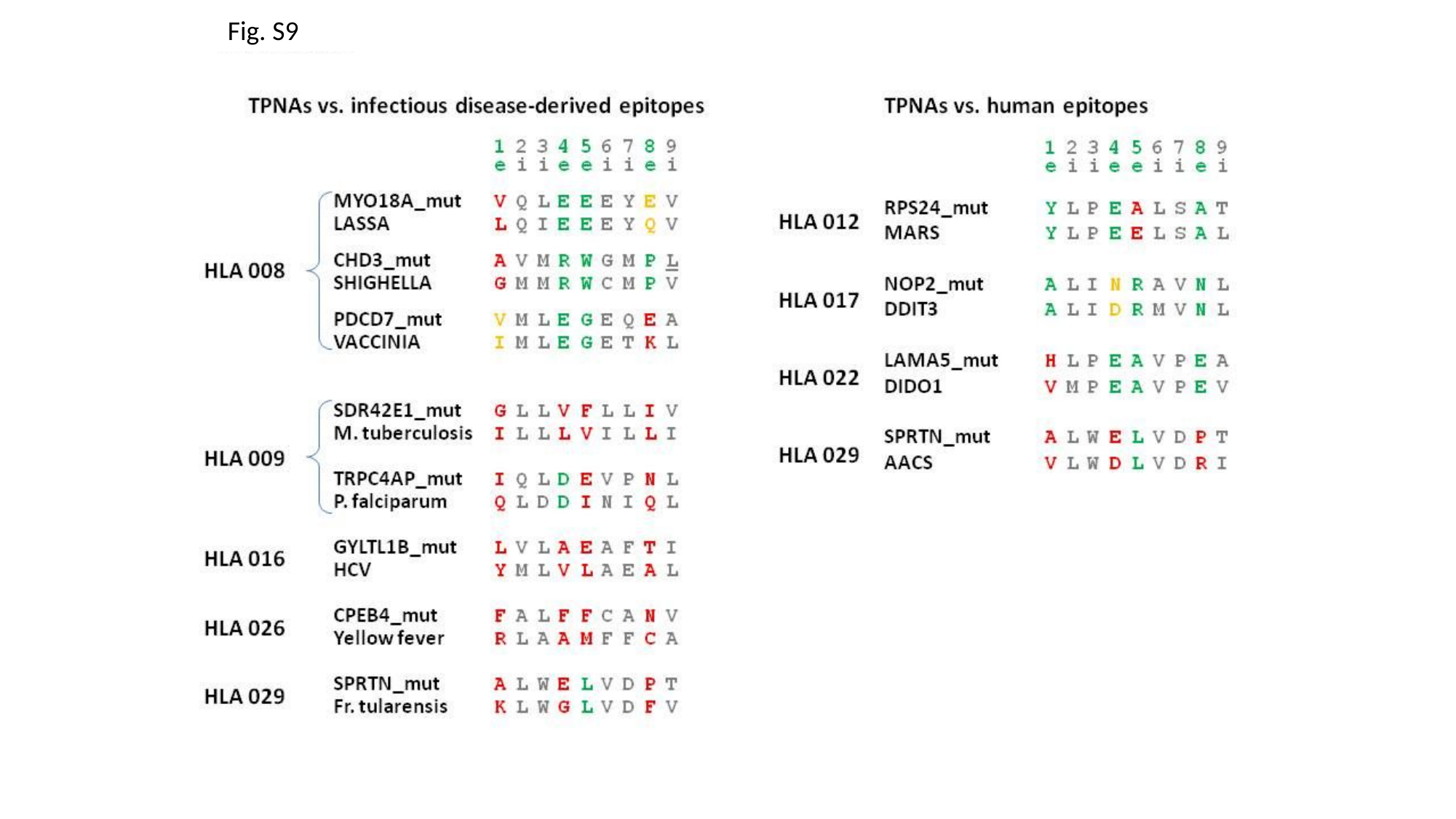

Fig. S9
